# Machine learning based algorithms for virtual early detection and screening of neurodegenerative and neurocognitive disorders: a systematic-review

**DOI:** 10.3389/fneur.2024.1413071

**Published:** 2024-12-09

**Authors:** Milad Yousefi, Matin Akhbari, Zhina Mohamadi, Shaghayegh Karami, Hediyeh Dasoomi, Alireza Atabi, Seyed Amirali Sarkeshikian, Mahdi Abdoullahi Dehaki, Hesam Bayati, Negin Mashayekhi, Shirin Varmazyar, Zahra Rahimian, Mahsa Asadi Anar, Daniel Shafiei, Alireza Mohebbi

**Affiliations:** ^1^Institute for Cognitive and Brain Sciences, Shahid Beheshti University, Tehran, Iran; ^2^Faculty of Medicine, Istanbul Yeni Yuzyil University, Istanbul, Türkiye; ^3^School of Medicine, Kermanshah University of Medical Sciences, Kermanshah, Iran; ^4^School of Medicine, Tehran University of Medical Sciences, Tehran, Iran; ^5^Student Research Committee, Ahvaz Jundishapur University of Medical Sciences, Ahvaz, Iran; ^6^School of Medicine, Ahvaz Jundishapur University of Medical Sciences, Ahvaz, Iran; ^7^School of Medicine, Shahid Beheshti University of Medical Science, Tehran, Iran; ^8^Master’s of AI Engineering, Islamic Azad University Tehran Science and Research Branch, Tehran, Iran; ^9^Department of Radiology, Shahid Beheshti University of Medical Sciences, Tehran, Iran; ^10^Department of Neuroscience, Bahçeşehir University, Istanbul, Türkiye; ^11^School of Medicine, Shahroud University of Medical Sciences, Shahrud, Iran; ^12^School of Medicine, Shiraz University of Medical Sciences, Shiraz, Iran; ^13^Student Research Committee, Shahid Beheshti University of Medical Sciences, Tehran, Iran; ^14^School of Medicine, Shahid Beheshti University of Medical Sciences, Tehran, Iran; ^15^Students Research Committee, Ardabil University of Medical Sciences, Ardabil, Iran

**Keywords:** neurodegenerative disorder, neurocognitive disorder, machine learning, early detection, AI

## Abstract

**Background and aim:**

Neurodegenerative disorders (e.g., Alzheimer’s, Parkinson’s) lead to neuronal loss; neurocognitive disorders (e.g., delirium, dementia) show cognitive decline. Early detection is crucial for effective management. Machine learning aids in more precise disease identification, potentially transforming healthcare. This comprehensive systematic review discusses how machine learning (ML), can enhance early detection of these disorders, surpassing traditional diagnostics’ constraints.

**Methods:**

In this review, databases were examined up to August 15th, 2023, for ML data on neurodegenerative and neurocognitive diseases using PubMed, Scopus, Google Scholar, and Web of Science. Two investigators used the RAYYAN intelligence tool for systematic reviews to conduct the screening. Six blinded reviewers reviewed titles/abstracts. Cochrane risk of bias tool was used for quality assessment.

**Results:**

Our search found 7,069 research studies, of which 1,365 items were duplicates and thus removed. Four thousand three hundred and thirty four studies were screened, and 108 articles met the criteria for inclusion after preprocessing. Twelve ML algorithms were observed for dementia, showing promise in early detection. Eighteen ML algorithms were identified for Parkinson’s, each effective in detection and diagnosis. Studies emphasized that ML algorithms are necessary for Alzheimer’s to be successful. Fourteen ML algorithms were discovered for mild cognitive impairment, with LASSO logistic regression being the only one with unpromising results.

**Conclusion:**

This review emphasizes the pressing necessity of integrating verified digital health resources into conventional medical practice. This integration may signify a new era in the early detection of neurodegenerative and neurocognitive illnesses, potentially changing the course of these conditions for millions globally. This study showcases specific and statistically significant findings to illustrate the progress in the area and the prospective influence of these advancements on the global management of neurocognitive and neurodegenerative illnesses.

## Introduction

Machine learning (ML) describes circumstances in which machines can mimic human minds in learning and analysis and thus be used to solve problems (1). Recent advances in ML have produced a computational framework by integrating a multitude of patient data and providing unique risk assessments and recommendations to each patient, which has the potential to revolutionize clinical decision-making (2) fundamentally.

Helping with diagnosis is one of the most significant uses of machine learning in this field. The promise of machine learning-based disease diagnosis (MLBDD), which is affordable and time-effective, is demonstrated by numerous researchers and practitioners (2). To identify chronic kidney disease, Ma et al. (2020) suggested a heterogeneous modified artificial neural network (HMANN) model that obtained an accuracy of 87–99% (3). To improve the diagnosis of COVID-19, Apostolopoulos and Mpesiana (2020) used a CNN-based Xception model on an imbalanced dataset of 284 COVID-19 and 967 non-COVID-19 patient chest X-ray images and achieved 89.6% accuracy in diagnosis (4). Regarding the diagnosis of diabetes, Yahyaoui et al. (2019) showed that the machine-learning RF technique works with an accuracy of 83.67% (5). The examples demonstrate how machine learning algorithms can provide more accurate and reliable disease diagnosis than other diagnostic techniques.

Neurodegenerative disorders are characterized by a gradual loss of neurons, often leading to death. The term covers a wide range of clinical diseases and progressive dementing conditions, including Alzheimer’s disease (AD), Parkinson’s disease (PD), and a number of other neurological disorders (6). Neurocognitive disorders, including delirium, mild cognitive impairment and dementia, are characterized by a decrease in cognitive functioning from a previously attained level (7). Many of these diseases are incurable and sometimes fatal, but early detection can significantly improve the ability to control them.

AD is the most prevalent form of dementia. Patients with AD have trouble remembering things, which limits their ability to learn. Due to the slow progression of AD and the difficulty of current diagnostic techniques in identifying it in its early stages, early diagnosis of the disease is crucial.

PD is a progressive and chronic neurodegenerative disease. The overall validity of PD’s clinical diagnosis, particularly in the early stages of the disease, is unsatisfactory (8).

Delirium is acute brain dysfunction that causes cognitive impairment and shifting attention. Numerous symptoms, such as significant psychomotor agitation, a low level of consciousness, or both, may be present. Traditionally, one or more physicians’ evaluations have been used to diagnose delirium clinically. However, this method of diagnosis might contain flaws because of the disease’s unstable nature (9).

As evident, standard clinical diagnostic techniques for neurodegenerative and neurocognitive diseases have flaws, which make it difficult and occasionally impossible to diagnose the disease, especially in its early stages. On the other side, machine learning algorithms can be highly accurate when it comes to diagnosing a variety of diseases. Recently, many studies have been conducted on the efficacy of ML algorithms as a quick and reliable alternative diagnostic method. Therefore, in this article, we aimed to systematically assess different uses of ML algorithms in detecting neurodegenerative and neurocognitive disorders early.

## Methods

This systematic review study was conducted as stated by Preferred Reporting Items for Systematic Reviews and Meta-Analyses (PRISMA2020) principles (10). This review has been registered on The Open Science Framework (OSF) (registration DOI https://osf.io/rtsyk/).

### Information sources, search strategy

A comprehensive search of several databases was conducted from inception to August 15th, 2023. The databases included PubMed/MEDLINE, Scopus, Google Scholar and Web of Science. As seen in table 1, the search for AI algorithms used for detecting and screening neurodegenerative and neurocognitive diseases involved a controlled vocabulary supplemented with keywords in each database. Table 1 demonstrates the specific search syntax used for each database involved.

**Table 1:** Search strategies and databases used in the study.

Database	Search strategy
PubMed	“deep learning”[Title/Abstract] OR “support vector machine”Title/Abstract] OR “Machine learning”[Title/Abstract] OR “supervised machine learning”[Title/ Abstract] OR “unsupervised machine learning”[Title/Abstract] OR “Machine learning”[MeSH Terms] OR “supervised machine learning”[MeSH Terms| OR “unsupervised machine learning”[MeSH Terms]) AND (“neurodegenerative disorders”[Title/Abstract] OR “neurodegenerative disease”[Title/Abstract] OR “neurodegenerative conditions”[Title/Abstract] OR “ALS”[Title/ Abstract] OR “Huntington’s disease”[Title/ Abstract] OR “Tauopathies”[Title/Abstract] OR “neurofibrillary tangles”|Title/Abstract] OR “myelitis”[Title/Abstract] OR “paraneoplastic polyneuropathy”|Title/ Abstract] OR “paraneoplastic cerebellar degeneration”|[Title/Abstract] OR “Tourette syndrome”|Title/Abstract] OR “neurofibromatoses”[Title/Abstract] OR “Encephalopathy”|Title/Abstract] OR “neuropathy”[Title/Abstract] OR “brain degeneration”[Title/Abstract] OR “CNS neurodegenerative disease”[Title/Abstract] OR “CNS degenerative disorder”[Title/ Abstract] OR “neurodegenerative diseases”[MeSH Terms] OR “neurocognitive diseases”[Title/Abstract] OR “neurocognitive disorders”[Title/ Abstract] OR “neurocognitive conditions”[Title/Abstract] OR “mild cognitive impairment”[Title/Abstract] OR “organic brain disorder” Title/Abstract] OR “acquired cognitive dysfunction”[Title/ Abstract] OR “dementia”|Title/Abstract] OR “delirium”|[Title/Abstract] OR “mild neurocognitive disorders”[Title/Abstract] OR “major neurocognitive disorders”[Title/ Abstract] OR “neurocognitive disorders”|MeSH Terms])
WOS	((TS = (“machine learning”)) OR TS = (“allgobrithem”)) OR TS = (“artificial inteligency”)| And ((TS = (detection)) OR TS = (“early detection”)) OR TS = (Diagnosis)) OR TS = (identification)) OR TS# (recognation)) OR TS = (observation)) OR TS = (“early diagnosis”) And ((((((((((((((((TS = (“Neurodegenerative disease*”)) OR TS-(ALS)) OR TS= (“Huntington”)) OR TS = (“Tauopathies and the subclassifications”)) OR TS= (Tauopathie*)) OR TS = (“Neurofibrillary tangle*”)) OR TS = (Myelitis)) OR TS= (“Paraneoplastic polyneuropathy”)) OR TS = (“Paraneoplastic cerebellar degeneration*”) OR TS = (“Tourette syndrome”)) OR TS = (Neurofibromatosis)) OR TS = (Encephalopathy)) OR TS = (Neuropathy)) OR TS = (“neurocognitive disorder* “)) OR TS = (“senile dementia”)) OR TS = (“Creutzfeldt-Jakob disease”)) OR TS = (“Diffuse Lewy body disease”)) OR TS = (“Multiple sclerosis”)) OR TS- (“Normal pressure hydrocephalus”)) OR TS = (“Pick disease*’)) OR TS = (amnesia)) OR TS = (“cognitive dysfunction”)) OR TS = (“consciousness disorder* “)) OR TS-(delirium)) OR TS = (dyslexia)
Scopus	TITLE-ABS-KEY (detection) OR TITLE-ABS-KEY (“early detection”) OR TITLE-ABS-KEY (diagnosis) OR TITLE-ABS-KEY (identification) OR TITLE-ABS-KEY (recognation) OR TITLE-ABS-KEY (observation) OR TITLE-ABS-KEY (“early diagnosis”)) AND (TITLE-ABS-KEY (“machine learning”) OR TITLE-ABS-KEY (“allgohrithem”) OR TITLE-ABS-KEY (“artificial inteligency”)) AND (TITLE-ABS-KEY (“Neurodegenerative disease”) OR TITLE-ABS-KEY (als) OR TITLE-ABS-KEY (“Huntington”) OR TITLE-ABS-KEY (“Tauopathies and the subclassifications”) OR TITLE-ABS-KEY (tauopathie) OR TITLE-ABS-KEY (“Neurofibrillary tangle”) OR TITLE-ABS-KEY (myelitis) OR TITLE-ABS-KEY (“Paraneoplastic polyneuropathy”) OR TITLE-ABS-KEY (“Paraneoplastic cerebellar degeneration “) OR TITLE-ABS-KEY (“Tourette syndrome”) OR TITLE-ABS-KEY (neurofibromatosis) OR TITLE-ABS-KEY (encephalopathy) OR TITLE-ABS-KEY (neuropathy) OR TITLE-ABS-KEY (“neurocognitive disorder “) OR TITLE-ABS-KEY (“senile dementia”) OR TITLE-ABS-KEY (“Creutzfeldt-Jakob disease”) OR TITLE-ABS-KEY (“Diffuse Lewy body disease”) OR TITLE-ABS-KEY (“Multiple sclerosis”) OR TITLE-ABS-KEY (“Normal pressure hydrocephalus”) OR TITLE-ABS-KEY (“Pick disease”) OR TITLE-ABS-KEY (amnesia) OR TITLE-ABS-KEY (“cognitive dysfunction”) OR TITLE-ABS-KEY (“consciousness disorder “) OR TITLE-ABS-KEY (delirium) OR TITLE-ABS-KEY (dyslexia))

### Data screening and eligibility criteria

We used the RAYYAN intelligent tool for systematic reviews to screen the search results (11). Titles and abstracts from 7,069 articles obtained from our search strategy were independently and blindly screened by six reviewers (Zh.M., Sh.K., H.D., A.A., H.B., M.Y.). The duplicate records were removed using the same tool. The conflicts were resolved by a seventh reviewer (Sh.K.) using RAYYAN’s compute rating feature.

### Inclusion criteria

The study was conducted on this specified list of neurodegenerative and neurocognitive diseases, and the search keywords included items below:

- Huntington
- Tauopathies and the subclassifications
- Neurofibrillary tangles
- Myelitis
- Paraneoplastic polyneuropathy
- Paraneoplastic cerebellar degeneration
- Tourette syndrome
- Neurofibromatoses
- Encephalopathy
- Neuropathy
- ALS
- Alzheimer’s disease (AD)
- Mild cognitive impairment (MCI)
- Parkinson’s disease (PD)
- Frontotemporal dementia (FTD)
- Lewy Body’s disease (LBD)
- Progressive supranuclear palsy (PSP)
- Corticobasal degeneration (CBD)
- Wernicke-Korsakoff syndrome
- Normal pressure hydrocephalus (NPH)
- Prion diseases, such as Creutzfeldt-Jakob disease
- Vascular dementia

Studies that were not available as open access were in languages other than English were conducted on animals, and were published as book chapters, Conference papers were excluded.

### Quality assessment of included studies

Two assessors (MY and HD) evaluated each study separately based on the Cochrane risk of bias tool, evaluating all included studies (12). With a focus on six domains—sequence generation, allocation concealment, blinding, incomplete data, and selective reporting—the Cochrane risk of bias tool is a widely used and standard tool that contains all the necessary questions to evaluate methodological quality and bias risk. The two assessors settled other biases and disagreements through discussion and consensus.

## Results

### Study selection

**Figure 1:**
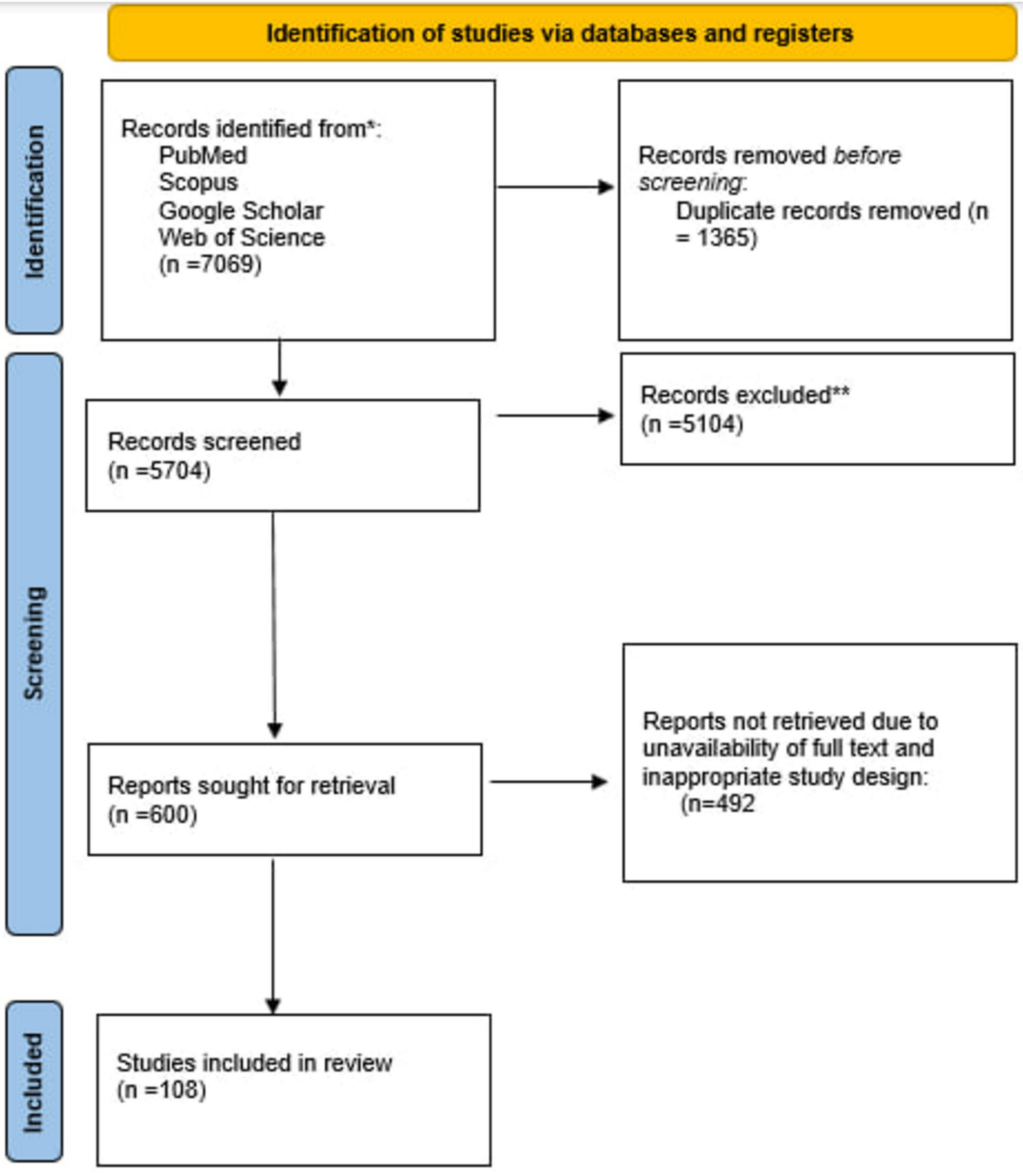
Flow diagram of the study selection procedure.

Our search strategies in four databases yielded 7,069 studies, 1,365 were eliminated as duplicates. At least two individuals screened each of 4,334 remaining studies through title and abstract. Unrelated studies whose full text was unavailable, did not meet our inclusion criteria, and were not in English were excluded. At last, 108 studies were included for interpretation. Figure 1 depicts the study selection procedure.

### Study characteristics

The included studies were published between 2015 and 2023. A study was carried out in Africa, another in Australia, 17 in Europe, 29 in America, and the remaining in Asia.

### Findings

In the included studies, 3,723,329 participants were examined. Thirty-four studies on AD, 14 on PD, 13 on MCI, 10 on dementia, 7 on MS and the remaining studies were carried out on other neurodegenerative and neurocognitive disorders.

### Dementia

In 10 studies conducted on dementia, 12 ML algorithms were used: XGBoost classification, Binary logistic regression (LR), A logistic model tree classifier combined with information gain feature selection, 3D convolutional neural networks (3D CNN), k-NearestNeighbor (kNN), support vector machine (SVM), random forest (RF), parallel recurrent convolutional neural network (PRCNN), support vector machine classifiers (SVC), support vector regression (SVR), partial least squares regression (PLSR) and Deep Neural Network (DNN), All of which showing promising results in early detection and screening of the disease. Table 2 summarizes our included studies.

**Table 2:** Summary of included studies on the machine learning algorithms for early detection of NCDs and NDDs.

Author	Year	Country	Aim of study	Population	Type of pathology	Used ML algorithm	Outcome	Conclusion
Raymond Gao et al. (28)	2023	USA	The present study aims to construct Polygenic Risk Scores (PRSs) for Alzheimer’s disease (AD) risk and Age at Onset (AAO). Additionally, it seeks to develop Machine Learning models for predicting AD risk and explore feature importance, including PRSs, conventional risk factors, and ICD-10 codes extracted from Electronic Health Records (EHRs).	457,936 participants	Alzheimer’s disease	XGBoost models	The study’s primary finding highlights the greater significance of Polygenic Risk Scores (PRSs) derived from Alzheimer’s disease (AD) risk and Age at Onset (AAO) compared to age alone in predicting AD. Additionally, the Machine Learning model identifies key predictors from Electronic Health Records (EHRs), such as urinary tract infection, syncope and collapse, chest pain, disorientation, and hypercholesterolemia, as crucial factors in the development of AD.	In conclusion, to tackle the broader issue of AD early detection, this study not only discovered critical traits for developing AD but also developed powerful explainable ML models.
Fayemiwo et al.(29)	2023	National Health and Aging Trends Study database	In this study, nine distinct experiments were conducted to determine which responses (either SP’s or proxy’s) in the “word-delay,” “tell-words-you-can-recall,” and “immediate-word-recall” tasks are essential in the prediction of dementia cases, and to what extent the combination of these two responses is helpful in the prediction of dementia.	National Health and Aging Trends Study (NHATS) was drawn from a nationally representative survey of Medicare recipients between the ages of 65 and older.	Alzheimer’s disease	Four ML algorithms (K-nearest neighbors (KNN), decision tree, random forest, and artificial neural networks (ANN)) were used	In the first scenario of experiments using “word-delay” cognitive assessment, the highest sensitivity (0.60) was obtained from combining the responses from both SP and proxies trained KNN, random forest, and ANN models. Also, in the second scenario of experiments using the “tell-words-you-can-recall” cognitive assessment, the highest sensitivity (0.60) was obtained by combining the responses from both SP and proxies trained KNN model. From the third set of experiments performed in this study on the use of “Word-recall” cognitive assessment, it was equally discovered that the use of combined responses from both SP and proxies trained models gave the highest sensitivity of 1.00 (as obtained from all the four models).	t can be concluded that the combination of responses in a word recall task as obtained from the SP and proxies in the dementia study (based on the NHATS dataset) is clinically useful in predicting dementia cases. Also, the use of “word-delay” and “tell-words-you-can-recall” cannot reliably predict dementia as they resulted in poor performances in all the developed models, as shown in all the experiments.
Bhandari et al.(30)	2023	India	This study employed explainable artificial intelligence (XAI) techniques to identify the significant set of gene features contributing to diagnosis and integrated gene expression data from various sources to diagnose Parkinson’s disease (PD) using Machine Learning (ML) based methods.		Parkinson’s disease		Based on the findings, it may be helpful to employ XAI when making early treatment decisions for Parkinson’s disease.	The study showcased a robust blood-based gene expression classification for Parkinson’s disease (PD) and healthy controls. By integrating PD datasets from various studies, the analysis gained reliability. Support Vector Machine (SVM) consistently outperformed other machine learning methods, and combining LASSO feature selection with Logistic Regression (LR) and SVM yielded the highest diagnostic accuracy.
Li et al. (31)	2023	USA	This research aims to explore machine learning techniques for early detection of Alzheimer’s disease (AD) and related dementias (ADRD) utilizing actual electronic health records (EHRs).	A total of 23,835 ADRD and 1,038,643 control patients	Alzheimer’s disease and related dementias	Various machine learning algorithms, including random forest and support vector machine, were initially tested on a smaller subset of the entire population. For subsequent experiments, logistic regression was chosen as the baseline, and Gradient Boosted Trees (GBTs), exhibiting the best performance in small-scale experiments, was selected for further analysis.	The best outcomes were obtained by the gradient boosting tree (GBT) models that were trained using the data-driven methodology and also a number of important clinical and sociodemographic factors were identified.	The study examined multiple cohorts of individuals with ADRD and associated dementias, identifying significant similarities and variability in predictions. The models that are being presented help identify ADRD early on and direct research and clinical trial recruitment.
Ostertag et al. (32)	2023	France	This study introduces a machine learning approach utilizing multimodal data (brain MRI and clinical information) from initial medical visits to predict long-term cognitive decline in patients.	229 subjects for training, 76 subjects for validation, and 76 subjects for testing	Alzheimer’s disease and Parkinson’s disease	This study introduces an adaptable deep neural network architecture for making long-term prognoses on the progression of neurological diseases, identifying high-risk individuals.	The model demonstrates effective long-term predictions of cognitive decline from any pair of early visits, even without a fixed time delay between them.	The model effectively predicts long-term cognitive decline with only two visits, accommodating irregular intervals. It also demonstrates successful knowledge transfer from Alzheimer’s to Parkinson’s, making it applicable to less studied diseases.
Ponce de Leon-Sanchez et al. (33)	2023	Mexico	The paper introduces a deep learning model, utilizing an artificial neural network with a single hidden layer, for predicting the diagnosis of multiple sclerosis.	99 with MS and 45 healthy controls	Multiple Sclerosis	K-Neighbors (KN), Gaussian Naive Bayes (GNB), C-Support Vector (CSV) Decision Tree (DT). Recursive Feature Elimination with Cross-Validation (RFECV) Deep Learning models Neural Networks	Feature selection was optimized based on accuracy, with the model achieving the highest accuracy using 35 features. The remaining 39 features were excluded, enhancing the efficiency of all compared classifiers.	Researchers propose an ANN model using 35 genetic features for MS diagnosis, outperforming conventional methods with high accuracy. The study underscores the potential clinical application of the ANN model in predicting MS based on genetic features, improving accuracy and enabling the emergence of new preventive treatments.
Russo et al. (34)	2023	Italy	The goal of the study was to develop a gait pattern involving particular spatial and temporal metrics that could be used to consistently differentiate between patients with Parkinson’s disease (PD) and those without mild cognitive impairment (MCI) through the use of supervised machine learning.	80 participants	Mild Cognitive Impairment and Parkinson’s Disease	Decision Tree (DT) Random Forest (RF) Naïve Bayes (NB) Support Vector Machine (SVM) K-Nearest Neighbor (KNN)	SVM and RF showed the best performance and detected MCI with an accuracy of over 80.0%.	The study demonstrates a robust relationship between gait dysfunction and Parkinson’s disease (PD)-related mild cognitive impairment (MCI). Notably, even on an independent dataset, selected gait parameters work well in machine learning methods for PD-MCI detection. By selecting homogeneous individuals, testing on an external patient group, and expanding the sample size, the research overcame earlier constraints to support these gait features as potential surrogate biomarkers for cognitive impairment in Parkinson’s disease (PD).
Syam et al. (35)	2023	India	The aim of this research was to propose a machine learning-based framework for accurate detection of Parkinson’s Disease (PD), Huntington’s Disease (HD), and Amyotrophic Lateral Sclerosis (ALS) from gait signals in both binary and multi-class detection environments.	?	Corticobasal Syndrome (CS), Huntington’s Disease (HD), Dementia, Amyotrophic Lateral Sclerosis (ALS), Progressive supranuclear palsy (PSP) and Parkinson’s Disease (PD)	The study proposes an ensemble framework named Ultaboost, utilizing Naïve Bayes and Logistic Regression, empowered by adaptive boosting principles such as Adaboost. Tested on prominent gait signal features obtained through feature selection techniques (IFS, ILFS, SFS), the ensemble framework addresses class imbalance with SMOTE.	In a multi-class environment, Infinite Feature Selection outperforms Infinite Latent Feature and Sigmis feature selection in detecting Parkinson’s and Huntington’s Disease from gait signal features	Using the UltraBoost ensemble framework, the paper presents a machine learning system that uses Naive Bayes and Logistic Regression to accurately detect Parkinson’s disease (PD), Huntington’s disease (HD), Amyotrophic Lateral Sclerosis (ALS), and Controls in binary and multi-class scenarios. Interestingly, the approach, which focuses on a small number of gait factors, performs well in binary classifications but has certain difficulties in multi-class environments, which are mostly related to class imbalance.
Tan et al. (36)	2023	Singapore	This study aims to develop a reliable machine learning (ML) model using socio-demographics, vascular risk factors, and structural neuroimaging markers for early diagnosis of cognitive impairment in a multi-ethnic Asian population.	911 participants	Cognitive Impairment	Logistic regression (LR), support vector machine (SVM), and gradient boosting machine (GBM).	The ensemble model demonstrated strong performance and it outperformed individual classifiers. Important predictors of cognitive impairment included age, ethnicity, highest education attainment, and neuroimaging markers.	The study demonstrates how machine learning techniques may be used to combine many data domains for precise early detection of cognitive impairment. In a population-based context, the model is scalable and makes use of characteristics that are easily accessible for the purpose of screening people who are at high risk of developing dementia.
Tayyab et al. (37)	2023	Canada	Using machine learning algorithms that can handle uncertain labels improves predictions when a substantial number of subjects have unknown outcomes in the dataset.	142 participants	Multiple sclerosis	Random Forest The study utilized three approaches, including a classic Random Forest (RF), to handle uncertain data points.	The Probabilistic Random Forest outperformed traditional Random Forest models, achieving the highest AUC.	In datasets with a significant number of subjects having unknown outcomes, employing machine learning algorithms that can model label uncertainty enhances predictive performance.
Tena et al. (38)	2023	Spain	The primary objective of this paper is to introduce a novel methodology for the early automated diagnosis of this dysfunction, surpassing the timing capabilities of clinicians.	45 ALS participants and 18 control subjects	Amyotrophic lateral sclerosis (ALS)	Five supervised classification models (RF, LR, LDA, NN, SVM) were implemented in R with standardized features using 10-fold cross-validation.	The Random Forest model achieved high accuracy, sensitivity, and specificity for classifying bulbar vs. control participants. Due to uncertainty in ALS patients without bulbar involvement, a semi-supervised SVM was used, resulting in improved performance. The model outperformed clinicians and existing methods, showcasing its efficacy in diagnosing bulbar dysfunction.	The obtained outcomes highlight the efficacy and feasibility of the approach suggested in this research. This strategy may lead to the development of an affordable and user-friendly instrument for the early identification and tracking of bulbar dysfunction in the early phases of the illness.
Mueller et al. (39)	2023	USA	The objective was to use electronic health records to find a risk estimation model for common delirium in patients being moved from emergency departments to inpatient units that would be therapeutically useful.	8,057 positive Delirium of total 28,531 participants	Delirium	Logistic Regression (LR), Decision Tree (DT), Random Forest (RF), Gradient Boosting Machine (GBM), Support Vector Machine (SVM), and K Nearest Neighbor (KNN).	Gradient Boosting Machine (GBM) had the best performance.	These findings will help design management or preventative measures.
Swarnalatha et al. (40)	2023	UAE	The current study aims to apply a novel deep feature that offers the best solution for EEG signal analysis and severity determination.		Alzheimer’s disease	A novel sandpiper-based recurrent neural system (SbRNS) has been developed to predict Alzheimer’s disease early stage.	The scheme that proposed by study had better performance that other same schemes.	Using EEG signals, a new SbRNS in MATLAB efficiently detects AD severity, exceeding traditional methods in terms of accuracy and performance.
Ahmed et al. (41)	2022	Egypt	The study suggests utilizing various modalities of Alzheimer’s disease brain images for the early identification of the disease in this publication.	300 individuals	Alzheimer’s disease (AD)	XGB CNN	The study achieved high accuracy, specificity and sensitivity in both early and late fusion.	The proposed model utilizes Laplacian Re-Decomposition for image fusion, combining data from MRI and PET modalities with XGBoost (XGB) to enhance early Alzheimer’s disease diagnosis, demonstrating superior performance compared to Naive Bayes (NB), Decision Trees (DT), Support Vector Machine (SVM), and Random Forest (RF) methods.
Ahmed et al. (42)	2022	Egypt	This paper’s primary goal is to detect and diagnose AD using SNP biomarkers that have a high degree of early classification accuracy.	1,569 subjects from 2 databases	Alzheimer’s disease	Boruta FS algorithm Gradient Boosting	The suggested method can be preferred for AD early detection.	
García-Gutierrez et al. (43)	2022	Spain	This paper has presented the design and implementation of a machine learning–based framework for the automatic diagnosis, especially, of neurodegenerative diseases.	329 patients	Alzheimer’s disease	EG or Bayesian classifiers	The results showed the potential of the framework for early and automated diagnosis with neuroimages and neurocognitive assessments from patients with Alzheimer’s disease (AD) and frontotemporal dementia (FTD).	The tool is presented from a XAI perspective to assist clinicians in diagnosis, as it offers all the necessary processes for analyzing these datasets, including feature selection via evolution, data preprocessing, and illness modeling.
Kavitha et al. (44)	2022	India, Iraq and Colombia	In this research, individuals affected by Alzheimer’s Disease are identified, and the objective is to detect individuals who may potentially have Alzheimer’s at an early stage.	MRI data from 150 patients aged	Alzheimer’s disease	Several techniques include Decision Tree, Random Forest, Support Vector Machine, Gradient Boosting, and Voting classifiers.	The suggested method shows excellent results, with a high average accuracy in validation.	Addressing Alzheimer’s involves risk reduction, early intervention, and accurate diagnosis. Future work will focus on improving detection techniques by extracting new features, eliminating irrelevant ones, and integrating metrics like MMSE and Education for enhanced accuracy.
Kumar et al. (45)	2022	India	The primary individual speech characteristics for dementia recognition are examined in this study. The primary contribution is the discovery of a small group of speech characteristics that improve the process of identifying dementia. The study also uses deep learning (DL) and machine learning (ML) models for efficient recognition.	A total of 442 subjects	Dementia	The study explores deep learning models, including artificial neural networks (ANN) CNN, RNN (GRU and LSTM), and a parallel recurrent convolutional neural network (PRCNN) for dementia recognition based on speech features.	Machine learning models are better than Deep learning models in diagnosing Dementia using speech characteristics.	The proposed approach shows promising results when compared to existing works on dementia recognition through speech analysis.
Li et al. (46)	2022	USA	In order to create feature subsets more effectively, the study presents the Ontology-guided Attribute Partitioning (OAP) approach, which considers domain-specific associations between features. These more effectively partitioned feature subsets are used in the study to create OAP-Ensemble Learning (OAP-EL), an ensemble learning framework.	276 very preterm infants	Cognitive Deficits	Ontology-guided Attribute Partitioning ensemble learning (OAP-EL) model	The proposed machine learning model had proficient results in early prediction of cognitive deficits at 2 years corrected age in very preterm infants.	The study introduced an advanced ensemble learning model for early predicting cognitive deficits in preterm infants, outperforming traditional methods. Future work includes exploring ontology-aided machine learning to understand brain features better.
Liu et al. (47)	2022	USA	The aim was to create and evaluate a precise deep-learning model for detecting adult hospitalized patients’ new-onset delirium.	A total of 331,489 CAM (confusion assessment method) assessments from 34,035 patients with 39,567 encounters were included in the final dataset.	Delirium	Logistic regression, random forest, support vector machine, and LightGBM models were developed with the training set and evaluated using 1,000-round bootstrapping on the testing dataset.	The LightGBM model showed the best performance, and by combining the LightGBM model with the LSTM, the model significantly improved performance. 20 features were identified using mean absolute SHAP values.	By combining the temporal trend-capturing abilities of LSTM with the LightGBM model’s predictive power, the model enhances the accuracy of predicting new-onset delirium.
Mehra et al. (48)	2022	India	The primary objective of this research is to precisely categorize people as either healthy or have Parkinson’s disease to create an efficient machine learning-based healthcare model. The most essential features for categorization are extracted with the primary goal in mind.	73 healthy subjects and 93 PD	Parkinson’s disease	The study utilizes a step regression-based approach for feature selection to enhance the classification of Parkinson’s disease (PD). The model is applied to three publicly accessible Parkinson’s datasets from diverse studies on Psyionet, all featuring Vertical Ground Reaction Force (VGRF) recordings from eight sensors under each foot.	The proposed model, incorporating effective pre-processing, feature extraction, and feature selection methods, achieved high accuracy when applied to three datasets.	This study introduces a superior machine learning-based Parkinson’s disease diagnosis model using wearable sensors.
Nelson et al. (49)	2022	USA	The method involves incorporating domain knowledge into clinical classifications by embedding individual patient data into a biomedical knowledge graph.	The study involved EHR data from 2,180,882 patients, patients with confirmed MS diagnoses 5,752 and control group (non-MS) consisted of 2,175,130.	Multiple Sclerosis (MS)	The Page rank algorithm was modified to embed millions of deidentified EHRs into a biomedical knowledge graph (SPOKE). This resulted in high-dimensional, knowledge-guided patient health signatures (i.e., SPOKEsigs) that were subsequently used as features in a random forest environment to classify patients at risk of developing chronic diseases.	The model successfully predicted the disease status of 5,752 subjects 3 years before their multiple sclerosis (MS) diagnosis	SPOKEsigs utilize Electronic Health Record (EHR) data to characterize patients clinically and biologically. The study demonstrates a clinical use case for detecting multiple sclerosis (MS) up to 5 years before the documented diagnosis, highlighting the distinctive biological features of the prodromal MS state.
Penfold et al. (50)	2022	USA	The goal was to create a natural language processing system and prediction model to identify Mild Cognitive Impairment (MCI) from clinical text.	4,185 patients from two datasets	Mild cognitive impairment (MCI) and	The study used a LASSO logistic regression approach to create a prediction model for MCI identification, incorporating NLP-derived concepts and demographic variables.	The prediction model showed modest performance in the validation dataset. Using a cutoff of 0.60, the classifier demonstrated low sensitivity and high specificity.	Though with low sensitivity, the model demonstrated a high negative predictive value, essential for population-based screening. It is comparable to widely used clinical tests.
Qiu et al. (51)	2022	Singapore	The study aimed to create a deep learning model, specifically a graph convolutional and recurrent neural network (graph-CNN-RNN), utilizing a series of brain structural MRI data to predict AD conversion based on age before clinical diagnosis.	2,489 subjects from two datasets.	Alzheimer’s disease	This study employed a deep learning longitudinal model, graph convolutional and recurrent neural network. (graph-CNN-RNN)	The graph-CNN-RNN accurately predicted the conversion of Alzheimer’s disease up to 4 years ahead of time with high reliability, and it consistently delivered accurate diagnoses of the condition at all periods.	The graph-CNN-RNN offered a detailed quantitative trajectory of brain morphology, from the early prognosis to the advanced stages of Alzheimer’s disease (AD).
Revathi et al. (52)	2022	India	The study aims to enhance prediction techniques and assess the cognitive function of individuals with potential dementia using the Cognitive Ability Test (CAT).	2,361 patients		This study utilized Support Vector Machine (SVM), Random Forest algorithm, and Multinomial Logistic Regression algorithm as machine learning techniques.	The first-stage classifier, using a Support Vector Machine (SVM), achieves a prediction accuracy of 86%, while the Random Forest classifier attains an accuracy of 71%. In the second stage, the Multinomial Logistic Regression algorithm for cognitive assessment achieves an accuracy of 89%.	The proposed work facilitates early prediction of individuals at risk of Alzheimer’s Disease through the analysis of clinical data.
Riad et al. (53)	2022	France and Belgium	The study aimed to predict clinical performance in Huntington’s Disease (HD), an inherited neurodegenerative disease, using machine learning applied to brief speech recordings.	103 individuals	Huntington’s disease	The study utilized the auto-machine-learning system, auto-sklearn, to predict clinical variables based on speech features. Auto-sklearn employs Bayesian optimization algorithms to identify the model with the optimal cross-validated performance on the training set.	Combining speech features with demographic information enabled the prediction of individual cognitive, motor, and functional scores.	In conclusion, this pioneering machine learning model and speech analysis accurately estimated classical scale scores for both pre-HD individuals and HD participants.
Schumann et al. (54)	2022	Germany	The study aimed to identify the optimal method for assessing fall risk by analyzing 11 gait datasets. It employed a new feature selection ensemble (FS-Ensemble) and four classification models—Gaussian Naive Bayes, Decision Tree, k-nearest Neighbor, and Support Vector Machine.	1,240 participants	Multiple Sclerosis	Gaussian Naive Bayes Decision Tree k-Nearest Neighbor Support Vector Machine (SVM)	The Gaussian Naive Bayes emerged as the most effective classification model for detecting falls across nearly all datasets.	The FS-Ensemble proved beneficial in enhancing classification models and is a suitable technique for reducing datasets with numerous features. Subsequent research focusing on additional risk factors, such as fear of falling, may offer further insights.
Sun et al. (55)	2022	China	A novel computation framework is introduced for predicting Multiple Sclerosis-associated miRNAs. The approach utilizes a network representation model to learn miRNA feature representations and employs a deep learning-based model for predicting Multiple Sclerosis-associated miRNAs.	102 MS-related miRNAs	Multiple Sclerosis	A convolutional neuron network (CNN)-based model is developed to integrate miRNA features and predict multiple sclerosis (MS)-related miRNAs. The workflow comprises a feature encoder, backpropagation training with dropout, and a Gaussian Naive Bayes (GaussianNB) classifier.	The assessment demonstrates that the proposed model accurately predicts miRNAs linked to Multiple Sclerosis, surpassing several existing methods by a significant margin.	The evaluation confirms that the proposed model outperforms existing methods and accurately predicts Multiple Sclerosis-related miRNAs.
Tufail et al. (56)	2022	China, Pakistan, Saudi Arabia, Canada	The objective of this study is to employ Convolutional Neural Network (CNN) architectures in both 2D and 3D domains, utilizing positron emission tomography neuroimaging, for the classification of early stages of Alzheimer’s Disease (AD) into AD, Mild Cognitive Impairment (MCI), and Normal Control (NC) classes.	AD 94, MCI 97 and NC 102	Alzheimer’s Disease	The study applied to transfer and non-transfer learning using 2D and 3D CNNs for binary. Custom 3D CNN architectures were used, and a transfer learning model based on Xception addressed MCI and AD classification.	3D-CNN architecture had the best performance. Data augmentation also contributed to superior performance in the multiclass classification task.	The outcomes support using deep learning models in the early diagnosis of Alzheimer’s disease.
Wang et al. (57)	2022	USA	This study introduces a novel Attentive All-level Fusion (AANet) system designed to integrate multi-level and multi-modality patient data (3D brain images, demographics, genetics, and blood biomarkers) into a deep-learning framework for early Alzheimer’s disease diagnosis.	11,333 valid MRI samples.	Alzheimer’s Disease	AANet incorporates a **Feature Pyramid Network (FPN)** for MRI image representation extraction and a self-attention fusion method to integrate features from various data modalities.	AANet demonstrated remarkable accuracy, surpassing various state-of-the-art methods. AANet presents an advanced methodological framework for disease diagnosis based on multiple modalities.	In summary, AANet exhibits remarkable potential for early Alzheimer’s Disease (AD) detection through its combination of the Feature Pyramid Network model and self-attention all-level fusion. It also provides a flexible framework for deep learning-based multi-modality and multi-level disease diagnosis.
Yu et al. (58)	2022	USA	The study aims to demonstrate an Alzheimer’s Disease (AD) diagnosis approach by utilizing the surface-enhanced Raman spectroscopy (SERS) fingerprints of human cerebrospinal fluid (CSF). The approach involves combining SERS with a convolutional neural network (CNN) for biomarker detection, specifically to analyze disease-associated biochemical changes in the CSF.	30 samples	Alzheimer’s Disease	A one-dimensional Convolutional Neural Network (CNN) was employed to process and classify surface-enhanced Raman scattering (SERS) spectral data.	An excellent correlation coefficient was observed between the test score and the Clinical Dementia Rating (CDR) score, indicating the feasibility of detecting Alzheimer’s Disease biomarkers through the innovative combination of Surface-Enhanced Raman Scattering (SERS) and machine learning.	In double-blind tests, a hybrid system combining Convolutional Neural Network (CNN) and Surface-Enhanced Raman Spectroscopy (SERS) demonstrated exceptional reproducibility, achieving 92% accuracy in diagnosing Alzheimer’s disease. Despite being based on a tiny sample size, the SERS neural network exhibits remarkable accuracy, making a biological test for Alzheimer’s diagnosis feasible. Future studies intend to increase patient sample sizes and investigate the possibilities of applying SERS/AI technology for clinical trial applications and early-stage diagnostics.
Zhang et al. (59)	2022	China	This study aims to explore potential peripheral blood biomarkers for the early diagnosis of PD.	ANIMALS we recused in this study.	Parkinson’s disease (PD)	SVM (Support Vector Machine) kNN (k-Nearest Neighbors) RF (Random Forest)	The study identified three upregulated genes in the peripheral-blood transcriptome datasets of Parkinson’s disease (PD) patients. Further analyses and validation in animal models revealed that SSR1 (Signal Sequence Receptor Subunit 1) was significantly upregulated in both models and negatively correlated with dopaminergic neuron survival.	In brief, this study identifies potential biomarkers for early PD diagnosis and establishes a potential artificial intelligence model for predicting Parkinson’s disease.
Valencia et al. (60)	2022	Spain	This study examines a technique for generating synthetic T1-weighted (T1-w) pictures from T2-FLAIR images. The study then evaluates the impact of utilizing original and synthetic T1-weighted images on the efficacy of the established approach for identifying longitudinal Multiple Sclerosis (MS) lesions.	136 subjects	Multiple Sclerosis	Fully Convolutional Network (FCN)	The proposed method can be useful.	The study demonstrates that synthetic images can effectively compensate for data scarcity or substitute for original images. This proves beneficial in standardizing contrast across diverse acquisitions, particularly in developing new algorithms for detecting T2 lesions in Multiple Sclerosis.
Adhikari et al. (61)	2021	Nepal	The transcripts of AD patients and control normal people were combined to build a novel dataset on low-resource language, namely Nepali, for this work and also provided baselines for the early identification of AD by utilizing a variety of machine learning (ML) and deep learning (DL) methods on a fresh dataset.	98 CN subjects and 168 AD patients	Alzheimer’s disease	Decision Tree (DT) K-Nearest Neighbors (KNN) Support Vector Machines (SVM) Naïve Bayes (NB) Random Forest (RF) AdaBoost XGBoost (XGB) CNN	best performing model is the attention-based CNN with domain-specific Word2Vec.	For conclusion, the study’s goal is to identify AD in Nepali speakers as soon as possible. This is a step in the right direction for resolving issues with disease identification and providing inspiration for future studies in this area. The main benefit of this automated system is that it predicts the existence of AD much more quickly.
Ahmed H et al. (62)	2021	Australia	This study aims to create multiple heterogeneous stacked fusion models by leveraging the strengths of various base learning algorithms, aiming to enhance the generalizability and robustness of machine learning models for Alzheimer’s disease diagnosis. The study combines written and spoken-based datasets to train the stacked fusion models.	1,598 AD patients and 1,628 healthy controls.	Alzheimer’s disease (AD)	Stacked Fusion Models Hybrid Stacked Fusion Model	Hybrid Stacked Fusion Model had better performance than Stacked Fusion Models.	This study recommends replacing the initial conventional screening test with such models that can be embedded into an online format for a completely automated remote diagnosis in light of the achieved performance and improved generalizability of such fusion models over single classifiers.
Etminani et al. (63)	2021	Italy, Belgium, Sweden, Switzerland, Slovenia, Germany and Netherlands.	This research aims to create and verify a 3D deep learning model using fuorine 18 fuorodeoxyglucose PET (18F-FDG PET) that can predict the final clinical diagnosis of Alzheimer’s disease (AD), dementia with Lewy bodies (DLB), mild cognitive impairment due to Alzheimer’s disease (MCI-AD), and cognitively normal (CN). The model’s performance will be compared to that of multiple expert nuclear medicine physicians.	757 patients	dementia with Lewy bodies, Alzheimer’s disease, and mild cognitive impairment	The 3D-CNN model (3D convolutional neural networks) is designed with reference to the architecture of VGG16.	The proposed model could achieve high levels of diagnosis and surpass human readers performance.	The final diagnosis of the most prevalent neurodegenerative diseases could be predicted by a 3D deep learning model with just the brain’s 18F-FDG PET, and it performed as well as human readers’ consensus.
Herzog et al. (64)	2021	UK	This study introduces a data processing pipeline that may be used with standard hardware. It analyzes structural alterations by using brain asymmetry parameters taken from MRI scans. Based on these parameters, the study uses machine learning to classify pathology.	MRI data of 750 subjects from ADNI	Alzheimer’s Disease	The pipeline tested various machine learning methods, focusing on brain asymmetry features enriched with Bag-of-Features for dementia diagnosis. Supervised learning was explored, including Naïve Bayes, Linear Discriminant, Support Vector Machine, and K-Nearest Neighbor. Transfer learning with AlexNet was also considered.	The introduced model was successful in distinguishing between normal cognitive, early mild cognitive impairment and AD patients.	In addition to providing a viable, affordable option for classifying dementia, the suggested pipeline may also prove helpful in treating other brain degenerative diseases that also cause alterations in brain asymmetry.
James et al. (65)	2021	USA	Can machine learning algorithms accurately predict 2-year dementia incidence in memory clinic patients, and how do these predictions compare with existing models?	1,568 dementia patients out of 15,307 samples	Dementia	The study implemented four machine learning algorithms, namely logistic regression (LR), support vector machine (SVM), random forest (RF), and gradient-boosted trees (XGB), for a classification task.	Machine learning algorithms outperformed two existing predictive models in predicting incident dementia within a 2-year timeframe.	It is indicated that machine learning algorithms can effectively predict the occurrence of dementia within 2 years.
Kleiman et al. (66)	2021	USA	The goal is to find optimized cognitive assessment features for detecting mild impairment improving routine screening.	1,565 participants from ADNI database	Alzheimer’s Disease	Developed a Multi-Classifier Network (MCN) by integrating multiple optimized random forest classifiers for three-class classification.	The proposed model achieved high classification level, and it is also able to detect in a short time.	The high detection rate and the minimal assessment time of the four identified features may be a practical starting point for developing screening protocols targeting cognitive impairment defined at CDR 0.5 and above.
Noh et al. (67)	2021	South Korea	This study seeks to employ machine learning (ML) to pinpoint significant features related to gait and physical fitness, aiming to predict a decline in global cognitive function among older adults.	306 patients	cognitive function decline in older adults	Eight machine learning models, namely SVM, DT, RF, NN, LASSO, EN, MCP, and SCAD, were employed to identify features with the lowest Root Mean Squared Error (RMSE) for both men and women.	Elastic Net selected five optimal features from the LP data for men, while Support Vector Machine selected twenty optimal features from the XI data for women. This approach successfully identified essential features for predicting a potential decline in global cognitive function in older adults.	The study successfully identified crucial features for predicting potential declines in global cognitive function among older adults. The proposed machine learning approach has the potential to inspire future research focused on early detection and prevention of cognitive function decline in the elderly.
Roshanzamir et al. (68)	2021	Iran	The study aimed to develop transformer-based deep learning models using natural language processing for the early risk assessment of Alzheimer’s disease by analyzing picture description test data.	170 AD patients and 99 healthy controls	Alzheimer’s disease	Logistic regression Single hidden layer neural network Single-layer bidirectional LSTM Three-layer CNN	The study evaluated models using picture description test transcripts from the Pitt corpus. That result improves 2.48% over the existing state-of-the-art.	Leveraging pre-trained language models enhances Alzheimer’s Disease (AD) prediction by addressing the challenges of limited datasets and diminishing the reliance on expert-defined features.
Sánchez-Reyna et al. (69)	2021	Mexico	This research introduces a novel methodology for creating a multivariate model that integrates various features to detect Alzheimer’s Disease (AD).	106 patients	Alzheimer’s disease	Subsequently, a support vector machine model was created to develop and validate the multivariate classification model.	A five-fold cross-validation showed an AUC of 87.63% for model performance, and in an independent blind test with 20 patients not considered during model construction, the final model achieved a perfect AUC of 100%.	The study introduces a methodology using genetic algorithms to select critical features from Alzheimer’s Disease (AD) data, including gene indexes and clinical assessments. These features are utilized to create supervised classification algorithms with SVM architecture. Model efficiency is assessed through cross-validation and a blind test, emphasizing high sensitivity and specificity for early AD detection among subjects with AD, MCI, or CN.
Singhania et al. (70)	2021	India, Saudi Arabia	The paper introduces a model utilizing biomarkers, including amyloid-beta protein, for detecting, predicting, and preventing Alzheimer’s Disease onset.	416 individuals for the cross-sectional MRI scan collection, and 373 individuals for the longitudinal MRI scan collection.	Alzheimer’s Disease	A Convolution Neural Network (CNN) based model is developed to predict AD at its early stages.	The findings demonstrated that the suggested model surpassed traditional Machine Learning (ML) methods, including Logistic Regression, Support Vector Machine, Decision Tree Classifier, and K Nearest Neighbor algorithms.	The model highlights the need for more research on biomarkers to improve algorithm accuracy in forecasting Alzheimer’s Disease progression and combines current patient circumstances to give preventive interventions.
Syed et al. (71)	2021	Pakistan and Australia	This study aims to propose a multimodal system capable of identifying linguistic and paralinguistic traits associated with dementia, serving as an automated screening tool.	?	Alzheimer’s dementia	Logistic regression (LRC), support vector machine classifiers (SVC), support vector regression (SVR), and partial least squares regression (PLSR) were used for the classification and regression tasks.	The system was evaluated on the Alzheimer’s Dementia Recognition Challenge dataset, achieving a new state-of-the-art performance in classification and matching the current state-of-the-art in regression.	Tested on the ADReSS challenge dataset, the system outperformed domain-knowledge-based features in audio and text and showed higher performance with deep neural embedding. In the ADReSS challenge, model ensembling was essential in setting a new state-of-the-art for classification and matching the existing state-of-the-art for regression. These results significantly advance the automated detection of Alzheimer’s disease.
Tsai et al. (72)	2021	Taiwan	The study suggests an intelligent assessment method for evaluating executive functions, employing machine learning to create an automated, evidence-based assessment model. Behavioral data is gathered by engaging participants in executive-function tasks within a virtual reality supermarket.	6 MCI or early AD participants and 6 control healthy participants	Cognitive impairment and Alzheimer’s disease	Logistic Regression, Support Vector Machines, Decision Tree, Random Forest, AdaBoost (Adaptive Boosting) and XGBoost (eXtreme Gradient Boosting) were applied.	The results indicated that the features derived from the Virtual Reality (VR) system strongly correlated with the diagnosis of individuals with MCI or early AD.	A virtual supermarket assessing executive functions was successfully tested on six healthy and six MCI/early AD participants. Trajectory analysis revealed 45 significantly different indices between groups. Machine learning achieved 100% accuracy in distinguishing healthy from MCI/early AD participants. Study limitations include a small sample size and technical challenges in VR computation. Future large-scale clinical trials are essential for validating the machine-learning model.
Uehara et al. (73)	2021	Japan	This study aims to conduct transcriptome analyses using SSL-RNAs and evaluate the potential of these expression profiles as diagnostic biomarkers for Parkinson’s disease (PD) through the application of machine learning.	65 PD patients and 65 control subjects	Parkinson’s disease	Extremely Randomized Trees (ERT)	Differential expression analysis identified over 100 genes differentially expressed between patients with PD and healthy controls in both cohorts, with upregulation of genes related to oxidative phosphorylation. Gene ontology analysis highlighted functional processes associated with PD.	The study explored the potential of utilizing SSL-RNA transcriptome for non-invasive differentiation between patients with Parkinson’s disease (PD) and healthy controls through machine learning.
Venugopalan et al. (74)	2021	USA	The study aims to employ deep learning (DL) for the integrated analysis of magnetic resonance imaging (MRI), single nucleotide polymorphisms (SNPs), and clinical test data, with the goal of classifying patients into distinct categories, including Alzheimer’s Disease (AD), Mild Cognitive Impairment (MCI), and controls (CN).	2004 patients	Alzheimer’s disease	Deep Learning Models like SVM, random forests, and decision trees.	Results demonstrate that deep models outperform shallow models, including support vector machines, decision trees, random forests, and k-nearest neighbors.	This study explores the potential of Deep Learning (DL) for improving the accuracy of Alzheimer’s Disease (AD) diagnosis and staging assessment using multi-modal data fusion. Key findings include the superiority of DL over shallow models for single-modality AD stage prediction, the effectiveness of a novel DL framework for multi-modality data fusion, and the application of perturbation and clustering-based feature extraction for interpretable DL model insights in AD stage prediction.
Wang et al. (75)	2021	USA and China	The study aims to develop and validate a deep learning model for detecting evidence of cognitive decline from clinical notes in the Electronic Health Record (EHR).	3,130 patients from two databases.	Cognitive decline	The study implemented a hierarchical attention-based deep learning structure along with four baseline machine learning algorithms: logistic regression, random forest, support vector machine, and XGBoost.	The deep learning model outperformed baseline models in both datasets.	A deep learning model proved to be accurate in this diagnostic study in identifying cognitive decline from clinical notes before diagnosing mild cognitive impairment (MCI). It performed better than other machine learning models and keyword-based searches, suggesting that it may be able to identify early cognitive decline in electronic health records (EHRs).
Zhu et al. (76)	2021	USA	This study aims to propose a flexible spatial–temporal solution that can predict the risk of Mild Cognitive Impairment (MCI) conversion to Alzheimer’s Disease (AD) before the onset of clinical symptoms. This is achieved by sequentially recognizing abnormal structural changes from longitudinal magnetic resonance (MR) image sequences.	151 subjects	Mild Cognitive Impairment (MCI) and Alzheimer’s Disease (AD)	Temporally Structured Support Vector Machine (TS-SVM) model	The early diagnosis method, utilizing only two follow-up MR scans, predicts conversion to Alzheimer’s Disease 12 months ahead of clinical diagnosis with an accuracy of 81.75%.	The paper introduces a novel method for predicting the conversion from Mild Cognitive Impairment (MCI) to Alzheimer’s Disease (AD) using only 2 MR images. The approach employs a Temporally Structural-SVM (TS-SVM) and joint feature selection framework. The model extracts partial MR image sequences at different time points, enforces monotony on SVM outputs, and achieves promising accuracy in classifying MCI converters and non-converters with fewer MR images compared to standard SVM approaches.
Mehmood et al. (77)	2021	China	The study aims to diagnose Alzheimer’s disease (AD) in its early stages by focusing on the problem with layer-wise transfer learning and brain imaging tissue segmentation.	85 NC patients, 70 EMCI, 70 LMCI, and 75 AD patients	Alzheimer’s Disease	Convolutional neural networks (CNNs)	The proposed method could distinguish between AD and NC.	Finally, comparing this model with comparable researches showed that it outperformed the most recent models in the field in terms of testing accuracy.
Montolío et al. (78)	2021	Spain	This study used clinical data and measures of retinal nerve fiber layer (RNFL) thickness using optical coherence tomography (OCT) to improve the diagnosis of multiple sclerosis (MS) and predict the progression of long-term impairment in MS patients.	104 healthy controls and 108 MS patients	Multiple Sclerosis	Multiple linear regression Support vector machine Decision tree K-nearest neighbors Naïve Bayes Ensemble classifier Long short-term memory	The ensemble classifier performed best in diagnosing MS, while LSTM performed best in the long-term prediction of MS disability course.	This study shows that it is possible to get an early MS diagnosis and predict the course of the disease by utilizing machine learning techniques with both clinical and OCT data.
Sudharsan et al. (79)	2021	India	This study aims to compare and recommend efficient classification methods for a specific and thoroughly analyzed dataset, with classifiers including SVM, significance vector machine, and RELM.	214 individuals	Alzheimer’s disease	Import Vector Machine (IVM) Regularized Extreme Learning Machine (RELM) Support vector machine (SVM)	RELM had the best accuracy among classifiers.	With limited data, the study addresses the diagnosis issues associated with Alzheimer’s/MCI, highlighting the efficacy of RELM and investigating ways to improve accuracy using item-measure methodologies.
Wang et al. (80)	2021	China	Through machine learning techniques and electronic health records, this study aimed to create a helpful tool to identify MS patients early.	Training set with 239 MS and 1,142 controls, and the test set with 23 MS and 92 controls.	Multiple Sclerosis	Extreme Gradient Boosting (XGBoost) Random Forest (RF) Naive Bayes K-nearest-neighbor (KNN) Support Vector Machine (SVM)	XGBoost had the best performance.	Reducing diagnostic delays in MS can be achieved by developing a diagnostic tool for early MS detection based on the XGBoost model and electronic health records.
Zeng et al. (81)	2021	China	This study propose a data-driven approach to identify potential biomarkers for Alzheimer’s disease (AD) and other poorly understood brain diseases.	458 subjects for training set and the 278 subjects for validation set.	Alzheimer’s Disease	Convolutional Neural Network (CNN) Ensemble Learning (EL) Genetic Algorithm (GA)	6 genes were identified in association with AD.	This method adaptively achieves more reliable and efficient candidate biomarkers in a data-driven manner, overcoming the limits related to the impact of subjective factors and dependence on prior knowledge.
Peng et al. (82)	2021	China	This research aimed to develop radiomics models using different machine learning methods to forecast the evolution of unenhanced Multiple Sclerosis (MS) lesions and identify the best model.	36 patients with MS	Multiple Sclerosis	three machine learning classifiers, including logistic regression (LR), random forest (RF), and support vector machine (SVM)	The best prediction performance was for the SVM classifier with ReliefF	The outcomes showed that the machine learning model based on radiomics could forecast how MS lesions will change over time.
Lee et al. (83)	2021	Republic of Korea	An novel strategy for identifying people with early-stage mild cognitive impairment (eMCI) is presented in this study. By concurrently learning functional connections from automatically selected areas of interest (ROIs) for each subject, the technique accounts for individual variability.	53 eMCI and 48 cognitively normal	MCI	Deep Neural Network(composed of a temporal embedding module, an ROI selection module, and a disease-identification module) Support vector machine (SVM) with FC computed using Pearson correlation. Self-attention conventional CNN LSTM-DG	The proposed model had efficacy In identifying eMCI.	This paper presented a novel framework for the identification of personalized early-stage mild cognitive impairment (eMCI), employing a Graph Convolutional Network (GCN) for relational representation learning and reinforcement learning for automatic ROI selection. The examination validated the efficacy of the approach in identifying pertinent areas documented in neuroscientific investigations on Alzheimer’s disease (AD) and moderate cognitive impairment (MCI).
Buegler et al. (84)	2020	EU/USA	Using just NMI (Neuro-Muscular Index) digital biomarkers, the comprehensive external validation study aimed to develop prediction models that were both widely applicable and dependable.	215 subjects for first dataset and 496 subjects for second dataset.	Dementia	XGBoost classification algorithm as the prediction model, using binary logistic regression	The proposed model was able to distinguish healthy subjects from subjects at risk to dementia within 3 years.	Digital biomarker prognostic models serve as beneficial resources for enormous scale population screening because they allow cognitive impairment to be detected early and allow for ongoing patient monitoring.
Alkhatib et al. (85)	2020	Lebanon	This study provides a detection algorithm that uses the load distribution during gait to categorize participants as either normal or Parkinson’s patients.	18 normal subjects and 29 PD subjects	Parkinson’s disease	Center of Pressure Path and Load Distribution	Using a linear decision boundary caused achievement of high classification accuracy.	This letter serves as a foundation for developing a portable device for real-time early detection of Parkinson’s disease. Additionally, it can be utilized for assessing the effectiveness of a rehabilitation program.
Gill et al. (86)	2020	Canada	The purpose of this study is to determine whether baseline mild behavioral impairment (MBI) status, which is utilized for NPS quantification, and brain morphological features are predictive of a follow-up diagnosis in individuals with normal cognition (NC) or MCI, median 40 months later.	102 individuals with NC and 239 with MCI	Dementia	A logistic model tree classifier combined with information gain feature selection was trained to predict follow-up diagnosis.	The optimal model required MBI total score and left hippocampal volume to classify participant as NC or impaired cognitive.	This study integrates clinical, neuropsychiatric, and MRI data using machine learning to predict future cognitive categories in non-demented older adults, showing improved accuracy with the inclusion of well-described neuropsychiatric symptoms.
Cheng et al. (87)	2020	China	The aim of this study is to overcome data density issues in understanding the Huntington’s disease (HD) mechanism by strategically reducing dimension size and employing machine learning to identify enriched pathways associated with HD using existing data.	157 HD and 157 controls	Huntington’s disease	Decision tree Rule induction Random forest Generalized linear model	66 potential HD-contributing genes were identified by proposed machine learning algorithms.	To further the pathophysiology of HD, the mutant HTT may obstruct the expression and trafficking of several identifiable genes.
Pan et al. (88)	2020	France	Determining which MCI individuals are at risk of developing AD-type dementia is the primary goal of this study.	1,005 subjects	Alzheimer’s disease	Multi-view Separable Pyramid Network (MiSePyNet) model was designed and also CNN algorithm was used.	Comparable to other state-of-the-art algorithms, the suggested technique can distinguish AD from Normal Control (NC). In terms of forecasting the course of mild cognitive impairment, the approach can outperform both conventional and deep learning-based algorithms.	The paper introduces MiSePyNet, a novel CNN model designed for AD prediction in the MCI stage and classification among NC subjects using 18F-FDG PET modality. MiSePyNet employs factorized convolution with separable CNNs for each view, achieving promising diagnostic results on ADNI data, especially for pMCI vs. sMCI.
Balea-Fernandez et al. (89)	2020	Spain	By analyzing sociodemographic, clinical, and analytical variables and choosing the best combination of them to accurately distinguish between subjects with major neurocognitive disorder (MNCD) and controls, this study seeks to develop a processing framework based on machine learning (ML) and optimization algorithms.	38 MNCD patients and 46 Controls	Dementia	Support Vector Machines (SVMs) Random Forest (RF) Artificial Neural Networks (ANNs) AdaBoost Ensemble Classifier (AB) Linear Discriminant Analysis (LDA) Logistic Regression (LR)	12 variables were identified in the validation set as the most relevant for MNCD diagnosis and Random Forest classifier had the best performance between used machine learning algorithms.	When used to comparatively limited preclinical and clinical data sets, machine learning (ML) is a promising method for automatic MNCD prediction. These findings provide credence to the idea that environmental factors play a role in the onset of AD.
Jauk et al. (90)	2020	Austria	The goal of the study was to put a machine learning algorithm that predicts delirium in hospitalized patients into practice and assess it. The intention was to identify high-risk individuals for prompt intervention both on admission and in the evening. Predictive performance and accuracy validation against expert assessments in a clinical environment were the main evaluation criteria.	cohort of 5,530 admissions of 4,663 patients	Delirium	Random Forest	The proposed model was successful at discriminating patients.	The study offers fresh insights into the integration of a machine learning algorithm into a clinical workflow and showcases its effectiveness in predicting delirium.
Liu et al. (91)	2020	China	The study introduces a novel framework for identifying EMCI using multi-modal data and graph convolutional networks (GCNs).	105 normal controls (NCs) and 105 subjects with early mild cognitive impairment (EMCI)	Early mild cognitive impairment	GCN model is adopted to perform the EMCI identification task.	The proposed method accurately identifies early cognitive impairment (EMCI) and outperforming some existing methods in this task.	This study proposed framework for identifying EMCIs is promising and effective for automated EMCI diagnosis in clinical settings.
Martynova et al. (92)	2020	Russia	This study focuses on identifying specific markers for multiple sclerosis (MS) diagnosis from a selection of 45 cytokines present in both serum and cerebrospinal fluid (CSF).	101 MS cases and 25 non-MS	Multiple Sclerosis	Five machine-learning models, namely k-Nearest Neighbor (KNN), Decision Tree (DT), XG Boost (XGB), Gaussian Naïve Bayes (gNB), and Random Forest (RF), were employed in this study.	Notably, the combination of CCL27, IFN-γ, and IL-4 among these biomarkers achieved the highest accuracy of 99% in MS diagnosis, indicating their significant role in MS pathogenesis	The study determined a specific group of cytokines in both serum and cerebrospinal fluid (CSF) that can serve for the diagnosis and categorization of multiple sclerosis (MS).
Pelka et al. (93)	2020	Germany	The aim of this paper is to fuse sociodemographic data such as age, marital status, education and gender, and genetic data (presence of an apolipoprotein E (APOE)-ε4 allele) with Magnetic Resonance Imaging (MRI) scans.	744 participants from 2 datasets	Mild cognitive impairment	LSTM based RNNs	Experimental results show that the proposed approach achieves 90% accuracy and 0.90 *F*_1_-Score at classification of aMCI vs. cognitively unimpaired participants on the HNR Study dataset, and 77% accuracy and 0.83 *F*_1_-Score on the ADNI dataset.	The study explores diverse fusion techniques for features from various medical modalities to enhance computer-aided diagnosis applications, particularly for classifying mild cognitive impairment. The approach has potential for adaptation to 3D deep learning methods, emphasizing multi-modal image representation.
Wang et al. (94)	2020	Saudi Arabia	This study intends to undertake a comparative analysis and provide insight on how well-performing advanced prediction systems work.	183 healthy individuals and 401 early PD patients	Parkinson’s disease (PD)	The methods include Deep Learning (DEEP) with a two-hidden-layer feed-forward neural network, Classification Trees (TREE), Boosting, Random Forest (RF), Logistic Regression (LOGIS), Discriminant Analysis (DIS), K-nearest Neighbor (KNN), and Support Vector Machines (SVM).	The designed deep learning model shows superior detection performance, outperforming other methods on average.	This study introduces a deep learning model for early Parkinson’s disease detection based on premotor features. The model achieves a high accuracy and outperforming twelve machine learning models. Despite the small dataset (584 individuals), the deep learning model demonstrates superior performance, showing promise for future applications as data size and complexity increase.
Zhu et al. (95)	2020	USA, China and Taiwan	The paper introduces a deep learning approach for dementia classification based on information gathered through informant-based questionnaires.	6,701 individuals	Senile Dementia	A Deep Neural Network (DNN) classification model was developed using the Keras framework. The model’s performance in discriminating between normal, MCI, VMD, Mild, Moderate, and Severe conditions was compared with other established classification models, including MLP, GCForest, random forest, AdaBoost, LogitBoost, Naïve Bayes, and SVM.	The Deep Neural Network (DNN) demonstrated superior stability and achieved the highest accuracy compared to seven conventional machine learning algorithms.	Accurately screening patients with a variety of cognitive conditions—such as normal cognitive function, mild cognitive impairment (MCI), very mild dementia (VMD), mild dementia (Mild), moderate dementia (Moderate), and severe dementia (Severe)—is made possible by the DNN classification model.
Magesh et al. (96)	2020	India	The objective of this study is to create a machine learning model that can accurately determine if a certain DaTSCAN signals Parkinson’s disease or not. The model also attempts to offer a clear justification for its predictions.	642 DaTSCAN SPECT images	Parkinson’s disease	Convolutional Neural Networks (CNN)	The proposed model distinguish PD from non-PD patients.	Utilizing a sizable and varied dataset, a machine learning model with remarkable accuracy and efficiency was created. By helping with the early diagnosis of Parkinson’s disease and fostering confidence in computer-aided medical diagnosis, this model helps healthcare professionals save time and resources.
Ghoreshi Beyrami et al. (97)	2020	Iran	The goal of this work was to develop a detection technique that would be reliable, affordable, and computationally efficient and that could be used to diagnose all three types of neurodegenerative diseases (NDDs).	16 healthy control subjects, 13 Amyotrophic lateral sclerosis (ALS), 15 Parkinson’s disease (PD), 20 Huntington’s disease (HD)	PD ALS HD	Sparse NNLS coding Support vector machine (SVM) Multilayer feed forward neural network (MLFN)	The proposed method could successfully diagnosis PD, ALS and HD with high accuracy.	The economical approach demonstrated resilience in a range of disease severity levels. This work developed an effective single-channel gait signal identification approach for three neurodegenerative diseases.
Roca et al. (98)	2020	France	The objective of this study was to create an algorithm that predicts the expanded disability status scale (EDSS) score for people with multiple sclerosis after two years by utilizing a variety of machine-learning techniques. The patient’s age, gender, and information from fluid-attenuated inversion recovery (FLAIR) magnetic resonance imaging (MRI) are the only factors used in this prediction.	1,446 subjects	Multiple Sclerosis	Convolutional Neural Network (CNN) Random Forest Regressors	The proposed algorithm was able to predict MS.	In summary, this approach, which only takes into account a single FLAIR sequence and some basic demographic data, assists in predicting the EDSS score at 2 years for MS patients.
Park et al. (99)	2020	South Korea	This paper aimed to develop a deep learning model that may predict Alzheimer’s disease (AD) by utilizing large-scale gene expression and DNA methylation datasets.	There were 2 datasets.(257 non-demented, 439 AD samples AND 68 normal and 74 AD samples)	Alzheimer’s disease	This paper proposes a deep neural network (DNN) model	The proposed model had better performance than conventional machine learning algorithms.	A multi-omics dataset was used to develop a novel algorithm for predicting Alzheimer’s disease. Compared to conventional dimension reduction techniques, the feature selection that took the biological context into account worked better. Furthermore, a prediction model based on deep learning showed improved performance.
Braga et al. (100)	2019	Portugal	The aim of this paper is to introduce a methodology for identifying early signs of Parkinson’s disease (PD) using free-speech analysis in uncontrolled background conditions.	Database1 = 22 PD Database2 = 30 healthy speakers Database 3 (test and validation set) = 28 PD patients	Parkinson’s disease	Random forest Neural Network (NN) Support vector machine	Random Forest (RF) or Support Vector Machine (SVM) have the potential to detect PD.	The study suggests a novel approach to Parkinson disease identification through free speech analysis. A short speech sample is subjected to an acoustic analysis by the system, which then utilizes the results to feed a classification algorithm built on machine learning. The outcome of the classification demonstrates the existence of acoustic indicators consistent with the speech characteristics of an individual with PD. Conversely, it may indicate that the provided recording exhibits a healthy pattern unrelated to the disease.
Abd El Hamid et al. (101)	2019	Egypt	The aim of this study is to identify the most significant SNPs linked to AD with improved classification accuracy and enhance early illness identification.	757 total subjects	Alzheimer’s disease	Sequential minimal optimization (SMO) algorithm with different kernels, Naive Bayes (NB), tree augmented Naive Bayes (TAN) and K2 learning algorithm have been applied.	Naive Bayes (NB) and K2 machine learning algorithms had the highest accuracy level.	By identifying important SNPs, this study attempts to increase the accuracy of Alzheimer’s disease (AD) diagnosis. The project aims to improve classification and early-stage detection by applying machine learning approaches to large genomic data sets from the ADNI-1 and WGS datasets.
Bi et al. (102)	2019	China	This paper presents analysis of EEG signals in time and frequency domain for detection of Alzheimer disease in early stage using Spectral and Complexity based features of EEG by use of suitable machine learning algorithm.	12 subjects	Alzheimer’s disease	Original model was created: Contractive Slab and Spike Convolutional Deep Boltzmann Machine (CssCDBM). But also there were several algorithms like: SVM and DBN.	The suggested approach outperforms other state-of-the-art techniques in terms of high-level representation extraction ability and yields superior outcomes.	In order to classify EEG spectrum images into three groups, the paper presents a cutting-edge discriminative deep probabilistic model with multi-task learning. This model surpasses several complex models and performs better than previous regularization methods for the early diagnosis of Alzheimer’s disease.
Maitra et al. (103)	2019	India	This research primarily aims to categorize individuals based on the presence or absence of early symptoms of a particular disease. Additionally, it explores the identification of subjects who may have progressed towards developing Alzheimer’s disease.		Alzheimer’s disease	A basic three-layer Neural Network architecture was trained using the OASIS longitudinal MRI dataset to distinguish between patients with early-stage Alzheimer’s disease and those without.	The highest accuracy achieved on the cross-validation set using the trained parameters is 86.49%, surpassing the performance of traditional learning algorithms previously applied to the same dataset. When focusing on the binary classification of ‘demented’ and ‘non-demented’ classes, a remarkable 100% accuracy was attained. Furthermore, perfect recall and a precision of 0.8696 were achieved specifically for the ‘demented’ class, indicating a high level of model performance in identifying individuals with dementia.	This study introduces novel features—socio-economic standing and educational background—and explores the role of gender in predicting Alzheimer’s disease. It offers a cost-effective approach by inferring missing MRI data through customized imputation methods. The research encourages a broader scope in feature exploration for disease prediction.
Pérez Del Palomar et al. (104)	2019	Spain	The study aims to compare axonal loss in ganglion cells detected through SS-OCT in the eyes of MS patients and healthy controls using various machine learning techniques. The goal is to assess the ability of these techniques to enhance the detection of damage in both the retinal nerve fiber layer (RNFL) and the complex Ganglion Cell Layer–Inner plexiform layer (GCL+).	79 MS and 179 healthy individuals	Multiple Sclerosis disease	A feed-forward neural network trained by a back-propagation algorithm multilayer perceptron (MLP) was tested. The support vector machine (SVM) tools were also analyzed. Finally, and taking into account that the data is very disperse among patients, the Adaptive Boosting (machine learning meta-algorithm) formulated by Freund and Schapire was used in conjunction with the above mentioned learning algorithms to improve performance.	Macular RNFL was introduced as robust predictor for MS while GCL+ did not contribute to MS prediction.	RNFL thickness measurements using SS-OCT effectively distinguish between healthy individuals and those with MS. Employing machine learning techniques based on these measures proves to be a reliable tool for aiding in the diagnosis of multiple sclerosis.
Petrone et al. (105)	2019	Spain	The study characterizes the signature of amyloid-positive subjects (PreAD) in comparison to Alzheimer’s disease (AD). The study aims to identify regions showing stage-specific changes in volume. These early structural changes in the brain are associated with disease progression, distinct from normal aging and later stages of AD.	79 controls, 50 preclinical AD (PreAD), and 274 MCI and dementia due to AD	Amyloid pathology	Machine learning is employed for voxel-wise prediction of amyloid-positive subjects (PreAD) within cognitively unimpaired individuals.	For classifying Controls (Ctrls) vs. Preclinical Alzheimer’s Disease (PreAD), the optimal follow-up time was determined to be Δt > 2.5 years and key brain regions demonstrating high discriminative power for detecting amyloid abnormalities included the medial, inferior, and lateral temporal lobes, precuneus, caudate heads, basal forebrain, and lateral ventricles.	The study suggests that applying machine learning to longitudinal brain volumetric changes enables highly precise prediction of amyloid abnormalities in cognitively unimpaired individuals. As a triaging method to identify amyloid-positive individuals, the longitudinal voxel-wise classifier is anticipated to eliminate unnecessary CSF and/or PET scans.
Prasad et al. (106)	2019	India	The study explores machine learning algorithms to enhance disease detection chances.	150 subjects	Alzheimer’s Disease	Decision Tree SVM Random Forest Adaboost	Random Forest had the highest accuracy.	Detecting and diagnosing Alzheimer’s disease is challenging with conventional clinical methods. Machine learning, although not flawless, offers a significant improvement in accuracy, making it a valuable tool in addressing these challenges.
Rehman et al. (107)	2019	England	The study aimed to utilize machine learning (ML) techniques to identify the optimal combination of gait characteristics for discriminating between individuals with Parkinson’s disease (pD) and healthy controls (Hc).	303 participants (119 PD, 184 HC)	Parkinson’s disease	Various models, including logistic regression (LR), linear discriminant analysis (LDA), k-nearest neighbor (KNN), classification and regression tree (CART), Naive Bayes (NB), support vector machine (SVM), random forest (RF), bagged decision tree (BDT), extra tree classifier (ETC), AdaBoost classifier (AC), gradient boosting classifier (GBC), and voting methods, were utilized for analysis.	Five clinical gait characteristics (mean step velocity, mean step length, step length variability, mean step width, and step width variability) were identified using Recursive Feature Elimination with Support Vector Machine (RFE-SVM) as accurate markers for classifying Parkinson’s disease (PD).	The study concluded by identifying a particular collection of gait characteristics that are essential for the precise early diagnosis of Parkinson’s disease (PD). These findings advance our knowledge of how machine learning methods may support well-informed healthcare decision-making.
Shen et al. (108)	2019	China	The objective was to create a novel deep belief network (DBN) framework utilizing 18F-fluorodeoxyglucose (FDG) positron emission tomography (PET) metabolic imaging. The goal was to identify individuals at the mild cognitive impairment (MCI) stage with pre-symptomatic Alzheimer’s disease (AD) and distinguish them from other MCI patients.	109 patients	Alzheimer Disease and Mild Cognitive Impairment	Deep Belief Network (DBN)	The approach used in the study shows promise as a potent tool for personalized precision medicine, particularly in predicting early Alzheimer’s disease (AD) progression within the population.	This study introduces a novel framework using a Deep Belief Network for early Alzheimer’s disease diagnosis. By extracting features from FDG-PET data and pre-extracting high-dimensional difference ROIs, the method surpassed multimodal approaches in distinguishing stable and progressive mild cognitive impairment. The network’s ability to learn discriminant features enhances classifier robustness, making it a powerful tool for AD diagnosis and prediction.
Spasov et al. (109)	2019	UK	The aim of this work is to present a novel deep learning architecture with a specific layer for 3D separable convolutions and dual learning. Finding individuals with mild cognitive impairment (MCI) who are more likely to acquire Alzheimer’s disease (AD) throughout a three-year period is the main objective.	192 AD patients, 409 MCI patients and 184 healthy controls	MCI AD	ANN models like batch normalization and dropout.	Proposed model could distinguish the MCI patients developing AD.	This study achieved a deep learning algorithm that incorporates baseline structural MRI, demographic, neuropsychological, and APOe4 genetic data from the ADNI database to predict the transition from mild cognitive impairment (MCI) to Alzheimer’s disease (AD) within a three-year timeframe. The obtained prediction performance shows high sensitivity, specificity, and accuracy.
Peña-Bautista et al. (110)	2019	Spain and France	The objective of the research was to develop and evaluate Artificial Neural Network (ANN) models by comparing them with both linear and non-linear models and analyzing their diagnostic efficacy for complex diseases.	Alzheimer’s disease (MCI-AD, *n* = 70) and healthy control participants (*n* = 26)	Alzheimer Disease	The diagnostic results achieved using an artificial neural network (ANN) model for each biological matrix were compared with those of the corresponding partial least squares (PLS) linear regression model and the non-linear support vector machine (SVM) models (radial and polynomial).	ANN models had better performance in comparison with PLS and SVM.	When the prognosis is complex, multidimensional and non-linear; lipid peroxidation and artificial neural networks (ANN) provide a valuable method for establishing a trustworthy diagnosis.
Saccà et al. (111)	2019	Italy	The purpose of this research was to determine which types of machine learning techniques performed best in differentiating Multiple Sclerosis patients from control participants and whether these techniques could be useful in supporting the early diagnosis of Multiple Sclerosis.	18 consecutive MS patients matched for demographic variables with 19 healthy controls	Multiple Sclerosis	Random Forest Support Vector Machine Naïve-Bayes K-nearest-neighbor Artificial Neural Network	Random Forest and Support Vector Machine had the best performance.	Based on the analysis, these results may indicate a promising first step toward a clinical diagnosis and prognosis.
Cai et al. (112)	2018	China	In this study, an enhanced fuzzy k-nearest neighbor (FKNN) method for the early detection of PD based upon vocal measurements was developed.	99 subjects	Parkinson’s disease	fuzzy k-nearest neighbor (FKNN) was the main algorithm that study used but there is also other algorithms that were discussed in the study like: Random forest and Support vector machine.	The proposed method had better performance than the other algorithms that is discussed in the study.	The method, called CBFO-FKNN, is an evolutionary instance-based approach incorporating chaotic BFO. The study’s findings indicated that the proposed CBFO-FKNN approach demonstrated superior performance compared to five other FKNN models.
Amoroso et al. (113)	2018	Italy	The suggested method seeks to identify new and effective PD markers for early diagnosis from MRI data.	374 PD and 169 NC	Parkinson’s disease	Random forest SVM classifier	The proposed model compares effectively with existing state-of-the-art MRI approaches.	This paper shows how complicated networks can be effectively used to create a unique brain connection model that offers precise markers for Parkinson’s disease (PD). When it comes to distinguishing between normal control (NC) and PD participants, the suggested methodology compares favorably to other cutting-edge methods.
Bairagi et al. (114)	2018	India	This research aims to improve the diagnostic accuracy of early-stage Alzheimer disease by analyzing EEG signals with an emphasis on spectral and wavelet properties.	100 participants	Alzheimer’s disease	Decision tree KNN Naive Bayes Support Vector Machine (SVM) Multilayer feed forward Neural Network	The use of spectral and wavelet increases classification accuracy.	This study concludes that the combination of spectral and wavelet characteristics yields higher classification rate and diagnostic accuracy and produces relatively better outcomes than using separate features.
Parisi et al. (115)	2018	New Zealand	The aim of this study was to explore the utility of a novel hybrid Artificial Intelligence-based classifier in facilitating early diagnosis of Parkinson’s disease (PD).	68 subjects	Parkinson’s disease	Multi-Layer Perceptron (MLP) Hybrid Machine Learning based algorithm	The proposed MLP-LSVM algorithm shows 100% accuracy and perfect area under the receiver operating characteristic curve, indicating swift convergence.	The hybrid MLP-LSVM model outperforms other classifiers, demonstrating superior accuracy, reliability, and computational speed in detecting Parkinson’s disease from speech data. It has potential for enhancing early diagnosis in clinical settings and improving the efficacy of automated speech processing systems.
Grassi et al. (116)	2018	USA	The current study aims to develop an algorithm for a 3-year prediction of conversion to AD in MCI and PreMCI subjects based only on non-invasively and effectively collectable predictors.	90 subjects with MCI and 94 subjects with PreMCI90 subjects with MCI and 94 subjects with PreMCI	Alzheimer’s Disease	The study employed machine learning techniques, including Elastic Net (EN), EN with polynomial features, Gaussian Processes (GP), k-Nearest Neighbors (kNN), and Logistic Regression(LR)	A Support Vector Machine had the best performance.	The developed algorithm demonstrates exceptionally high cross-validated performance, surpassing the majority of existing algorithms.
He et al. (117)	2018	USA	An artificial neural network (ANN) framework is proposed for early prediction of cognitive deficits in very preterm infants using functional connectome data derived from resting-state fMRI.	28 very preterm infants	cognitive deficits/function in individual very preterm infants	The study employs a Stacked Sparse Autoencoder (SSAE), a type of artificial neural network (ANN), for high-level connectome feature learning. For outcome prediction, the study utilizes a Support Vector Machine (SVM) classifier, a supervised classification method based on decision planes. In this case, a SVM classifier with a linear kernel is employed for predicting outcomes.	The ANN successfully predicted cognitive outcomes at 2 years of corrected age.	The study used neonatal-optimized methods to construct functional brain connectomes in very preterm infants. The findings suggest the potential of functional brain connectome data as prognostic biomarkers and demonstrate the feasibility of using artificial neural networks to capture individual variability. Further validation in a larger study is recommended.
Schütze et al. (118)	2018	Brazil	The aim of the study was to explore the correlation between cognitive performance and distinct resting-state brain metabolism patterns in individuals with NF1.	16 individuals with NF1 and 16 individuals for control	Neurofibromatosis type 1	Gaussian Processes (GP) regression, implemented with the Gaussian Process regression (GPR) within the kernlab R library, was used for the analysis. The linear kernel function “Vanilladot” was selected. Additionally, principal component analysis (PCA) was applied to address overlapping neuropsychological test data, and leave-one-out cross-validation (LOOCV) was used for accuracy assessment.	In the NF1 study, cognitive differences were found in intelligence, memory, and motor skills compared to controls.	The study demonstrated accurate prediction of neuropsychological performance from brain metabolism in NF1 patients using Gaussian Processes. This suggests an underlying metabolic pattern associated with global/verbal aspects of cognitive functioning in this group.
Oh et al. (119)	2018	South Korea	The purpose of this research was to investigate the potential of using heart rate variability (HRV) in combination with machine learning techniques to successfully differentiate between patients who were delirious and those who were not.	140 patients in ICU		Normalized mutual information feature selection(NMIFS) linear support vector machine (SVM) SVM with radial basis function (RBF) kernels. Linear extreme learning machine (ELM) ELM with RBF kernels. Linear discriminant analysis (LDA) Quadratic discriminant analysis (QDA).	SVM with radial basis function (RBF) kernels had the best accuracy in detecting delirium.	Successful classification was made possible by the identification of detectable heart rate variability (HRV) patterns in delirious patients. The study indicates the possibility of anticipating or identifying delirium in the intensive care unit (ICU) at an early stage by combining these patterns with machine learning.
Lu et al. (120)	2018	Canada	The goal of this research is to present a novel deep learning framework that uses FDG-PET metabolism imaging to identify and differentiate presymptomatic AD patients from other MCI subjects who are in the Mild Cognitive Impairment (MCI) stage.	1,051 subjects	Cognitive Impairment (MCI) stage and Alzheimer’s disease	Deep Neural Network (DNN) algorithms like Unsupervised Pre-training, Supervised Fine-tuning, and Dropout Strategy.	The advanced DNN model had high accuracy of classification using FDG-PET metabolism data which had better performance in comparison with same type of studies.	This research uses multiscale patch-wise FDG-PET deep-learning characteristics to present a novel framework for early diagnosis of Alzheimer’s disease. It uses ensemble classifiers and transfer learning to improve the ability to distinguish between people with mild cognitive impairment who are stable and those who are improving.
Mishra et al. (121)	2017	USA	The brain regions where tau PET provide the most useful information in differentiating between low and high levels of [18F]-AV-1451 binding were identified using an unsupervised learning, data-driven technique.	84 cognitively normal (CN), and 13 dementia due to Alzheimer’s disease	Alzheimer Disease	Sparse K-means	The study identified key regions, including the entorhinal cortex, amygdala, lateral occipital cortex, and inferior temporal cortex, using FreeSurfer. These regions were effective in distinguishing between different groups. The study also suggests using an AV-1451 SUVR cut-off of 1.25 to define high tau levels based on imaging data.	The summary metric was further confirmed in a cohort of thirteen patients with Alzheimer’s disease, and among the diseased population, β-amyloid PET imaging was found to be correlated with cognitive failure.
Pereira et al. (122)	2017	Portugal	This study suggests a Time Windows approach to address the MCI-to-AD conversion issue.	719 MCI patients AND two independent cohorts (604 for cross- validation (CV) set and 115 for independent validation set)	Mild cognitive impairment and Dementia	Naïve Bayes, Decision Tree, Random Forest, Gaussian, and Polynomial-kernel Support Vector Machines, k-Nearest Neighbor, and Logistic Regression algorithms were used.	The Time Windows approach demonstrated superior performance compared to the First Last approach. The study achieved successful prediction of dementia conversion as early as 5 years before the event.	Time window-based prognostic models outperform common approaches for predicting MCI to dementia progression, offering enhanced clinical relevance by specifying conversion within a temporal interval, enabling timely interventions.
Zhao et al. (21)	2017	USA	To explore the value of machine learning methods for predicting multiple sclerosis disease course.	1,693 participants	Multiple Sclerosis	Support vector machines (SVM) were used to build the classifier, and compared to logistic regression (LR) using demographic, clinical and MRI data obtained at years one and two to predict EDSS at 5 years follow-up.	In summary, baseline data alone had limited predictive value for disease progression in multiple sclerosis (MS). Clinical observation for 1 year improved predictive accuracy, and the addition of 1 year MRI data further enhanced sensitivity and specificity. The use of non-uniform misclassification costs in the support vector machine (SVM) model, favoring increased sensitivity, significantly improved predictions.	
Peng et al. (123)	2017	China	The study’s objective is to provide a machine learning technique for the identification of morphometric biomarkers in Parkinson’s disease that is based on multilayer region-of-interest (ROI) characteristics.	69 PD patients and 103 normal controls	Parkinson’s disease	multi-kernel SVM classifier	The proposed method was able to distinguish PD patients from normal controls with good performance.	The suggested approach confirms the method’s promise for assisted disease diagnosis by demonstrating a promising detection capability for morphometric biomarkers in Parkinson’s disease.
Zhu et al. (124)	2016	USA	The aim of the study is to introduce a flexible spatial–temporal solution for the early detection of Alzheimer’s Disease (AD) by identifying abnormal structural changes from longitudinal MR image sequences.	150 subjects	Alzheimer’s Disease	Since magnetic resonance (MR) image is non- invasive and widely used in clinic practice, we present a novel temporally structured SVM (TS-SVM) on longitudinal MR image sequences.	The study demonstrates that the proposed method can predict Alzheimer’s Disease (AD) conversion 12 months prior to clinical diagnosis.	In this study, a temporally structured Support Vector Machine (SVM) based innovative early Alzheimer’s Disease (AD) detection technique is introduced. The method enforces monotony on SVM output, reflecting the irreversible nature of AD progression, to achieve constant and accurate classification results. The classification margin is changed for early AD identification in order to boost confidence with additional follow-up scans. To maximize the compatibility between chosen features and trained classifiers, the approach also includes combined feature selection and TS-SVM training.
Cabral et al. (125)	2015	Portugal	This research aims to explore the effects of disease stage on machine learning approaches’ conversion prediction capabilities.	100 MCI patients	MCI	Support Vector Machines (SVM) Gaussian Naive Bayes (GNB)	It was revealed that MCI to AD conversion can be predicted as early as 24 months prior to conversion.	Using FDG-PET scans at various prodromal stages, this study studied the change of MCI to AD. This study classified participants as MCI-NC and MCI-C using longitudinal neuropsychological test scores (CDR and MMSE) from the available individual duals identified as MCI. Furthermore, the duration of conversion for the MCI-C subjects was calculated.
Moradi et al. (126)	2015	Finland, France and Germany	The study introduces an innovative magnetic resonance imaging (MRI)-based approach for forecasting the conversion from Mild Cognitive Impairment (MCI) to Alzheimer’s Disease (AD) one to 3 years before the clinical diagnosis.	AD (Alzheimer’s disease) with 200 subjects, NC (normal cognitive) with 231 subjects, sMCI (stable MCI) with 100 subjects, pMCI (progressive MCI) with 164 subjects, and uMCI (unknown MCI) with 100 subjects.	Alzheimer’s disease and Mild cognitive impairment	The study employs a two-stage classification framework. In the first stage, a regularized logistic regression framework selects informative MRI voxels, avoiding the use of MCI data for feature selection. The second stage trains a semi-supervised LDS classifier, integrating unlabeled uMCI samples to fine-tune decision rules. The LDS algorithm, operating on machines like transductive support vector machines, combines a graph-distance kernel.	The proposed model which is using MRI images has the potential of detecting AD in early stage.	The study’s conclusions underscore the importance of MRI in predicting MCI-to-AD conversion and the promise of the suggested method for early Alzheimer’s disease (AD) diagnosis. Crucially, the findings show that combining MRI data with the outcomes of cognitive tests improves the prediction of MCI-to-AD conversion.
Suk et al. (127)	2015	USA and Republic of Korea	The paper introduces a deep learning approach using a stacked auto-encoder (SAE) to create a latent feature representation. It posits the presence of intricate patterns within low-level features and emphasizes that incorporating latent information enhances the robustness of the model in the classification of Alzheimer’s Disease (AD) and Mild Cognitive Impairment (MCI), leading to high diagnostic accuracy.	51 AD patients, 99 MCI patients and 52 HC(health control) subjects	Alzheimer’s disease (AD) and mild cognitive impairment (MCI)	stacked auto-encoder (SAE)	The results suggests that deep learning offers valuable insights for neuroimaging data analysis, particularly in the diagnosis of brain diseases.	The study suggests that deep learning has the potential to provide novel insights into neuroimaging data analysis, and the paper introduces the application of this method to the diagnosis of brain diseases for the first time.
Maestú et al. (128)	2015	USA and Spain	The objective of the project was to use functional connectivity measurements and magnetoencephalography (MEG) to individually separate people with mild cognitive impairment (MCI) from people who are aging normally.	102 MCI patients and 82 age- matched controls	Mild Cognitive Impairment	Random Forest Bayesian Network C4.5 induction tree K-nearest Neighbor Logistic Regression Support Vector Machine	The propose non-invasive model may help to detect dysfunction in CNS.	The article proposes a novel blind analysis using machine learning discriminating techniques on data collected from multiple places. Using patterns of functional connectivity determined by magnetoencephalography (MEG), the study is able to successfully identify patients with mild cognitive impairment (MCI).
Mandal et al. (129)	2014	India	The primary goal of this study is to enhance the accuracy and reliability of Parkinson’s disease (PD) diagnosis, aiming to prevent the misdiagnosis of patients.		Parkinson’s disease	Neural networks, Support vector machines, LogitBoost, AdaBoost M1, Furia, Ensemble selection, Pegasos, Rotation forest, Bayesian logistic regression, Sparse multinomial logistic regression and SMLR component wise update rule.	The main finding relates to the incorporation of logistic regression and Haar wavelets in Random Forests (RFs), which improves the classifiers capacity for predictive analysis.	The software reliability and quality of the computer-aided diagnosis system have advanced significantly in this work, which also provides the best experimental results with statistical inference that supports them.
Mundt et al. (130)	2000	USA	A brief dementia screening questionnaire has been developed and validated. This tool is intended for broad administration in the general population.	272 patients extracted from the dementia screening	Alzheimer’s Disease	This algorithm, similar to classification and regression tree analysis, creates a binary tree by iteratively dividing data into more homogeneous subsets.	The results of this analysis were used to help four dementia experts create a dementia screening instrument amenable to application and scoring by nonclinical personnel and also the proposed model was successful in distinguishing healthy controls from possible AD patients.	The empirically developed scale’s psychometric characteristics and ability to distinguish between control subjects and probable or possible Alzheimer’s patients suggest that it has great potential for use as a dementia screener for the general public.
Velazquez et al. (131)		USA	This research centers on delivering personalized predictions for the transition from Mild Cognitive Impairment (MCI) to Alzheimer’s Disease (AD) using a balanced random forest model that utilizes clinical data.	383 Early Mild Cognitive Impairment (EMCI)	Alzheimer’s disease and early mild cognitive impairment	Random Forests are an ensemble learning method used for classification and regression.	The study found that a random forest model demonstrated effectiveness in predicting the conversion of Early Mild Cognitive Impairment (EMCI) patients to Alzheimer’s Disease (AD) using clinical features.	In practical terms, proposed model has the potential to be a valuable tool in clinical settings. It can be used to forecast the progression to Alzheimer’s Disease from an early stage or pinpoint individuals suitable for participation in clinical trials.
Minhad et al. (132)		USA	Making an accurate distinction between those who are healthy and those who have dementia is the main goal. Enabling the early diagnosis of dementia development is the ultimate goal. Thus, it is clear that activity prediction algorithms have promise for early dementia symptom detection without the need for costly diagnostic tests.		Dementia	This study introduced SPADE, a novel model which uses Decision tree algorithm.	SPADE achieved better results than other same models like SPEED\M-SPEED.	Thus, it is clear that activity prediction algorithms have potential for the early detection of dementia signs without the need for costly clinical procedures.
Ortiz et al. (133)		Spain	In order to diagnose Alzheimer’s disease (AD) and early AD, the research presents a Deep Learning method called Deep Belief Networks (DBN), which combines structural and functional imaging data. It makes use of an ensemble of DBNs that have been trained on data from various brain areas.	68 NC, 70 AD, 111 MCI and 26 Late MCI	Alzheimer’s Disease (AD) Mild Cognitive Impairment (MCI)	Deep belief networks Support vector machines	SVM with Deep Belief Networks (DBN-SVM) yielded the best results. However, the most successful approach was using DBNs as feature extractors in FEDBN-SVM, outperforming other methods including PCA and SVM ensemble (SVM-e).	
Prashanth et al. (15)		India and USA	This paper aims to classify early-stage Parkinson disease (PD) subjects using non-motor features of rapid eye movement behavior disorder (RBD) and olfactory loss combined with important biomarkers such as measurements of the cerebrospinal fluid (CSF) and markers associated with dopaminergic imaging.	183 healthy normal and 401 early PD subjects	Parkinson’s disease	simple (Naïve Bayes and logistic regression) and advanced (SVM, random forests and boosted trees) classifiers	The best performance was for SVM classifier.	The study suggests that non-motor, CSF, and imaging markers together could help with preclinical PD diagnosis.

SVM and XGBoost are prominent models for early dementia detection, each with distinct advantages and disadvantages regarding sensitivity and specificity. SVM excels in handling unbalanced datasets, achieving high sensitivity and specificity (over 90% in some studies), making identifying subtle early-stage symptoms practical. However, it can struggle with scalability and requires significant computational resources. In contrast, XGBoost offers flexibility and speed, handling various input features well, with sensitivities reaching between 80 and 85%. Yet, it may only perform in specificity compared to SVM if carefully tuned, which demands advanced cross-validation methods and more computational power. Both models demonstrate effectiveness; however, SVM offers enhanced specificity, which is vital for precise diagnostic accuracy. However, XGBoost excels in sensitivity but necessitates meticulous tuning to achieve optimal performance.

### Parkinson’s disease

The ML algorithms used for PD are as follows: Center of Pressure, Load Distribution, Random forest algorithm, Neural Network (NN), Support vector machine, and affine registration using the FSL library developed by the Oxford Centre for Functional MRI of the Brain (FMRIB), Multi-Layer Perceptron (MLP), Vertical Ground Reaction Force (VGRF), logistic regression (LR), linear discriminant analysis (LDA), kNN, classification and regression tree (CART), Naive Bayes (NB), bagged decision tree (BDT), extra tree classifier (ETC), AdaBoost classifier (AC), gradient boosting classifier (GBC), Extremely Randomized Trees (ERT), Discriminant Analysis (DIS), Deep Learning (DEEP). All mentioned algorithms showed significant early PD detection, diagnosis and screening capabilities, and most had considerable sensitivity and specificity.

According to the review of studies, various algorithmic models have been employed for the early diagnosis of Parkinson’s disease, with deep learning models demonstrating exceptional effectiveness. These models achieve nearly 100% accuracy, along with high sensitivity and specificity. Their advantages include remarkable accuracy, non-invasive techniques utilizing medical imaging data, and automated feature extraction, which minimizes the need for manual data handling. However, deep learning models necessitate substantial computational resources and large volumes of labeled data, and their “black box” nature poses challenges for interpretability.

### Alzheimer’s disease

In the included studies, much attention was paid to using ML algorithms for diagnosing and progressing Alzheimer’s. The following algorithms were used for detection, screening and progression of AD, all of which were successful for the purposes: Sequential minimal optimization (SMO), Naive Bayes (NB), tree augmented Naive Bayes (TAN), K2, MATLAB PatternRecognition toolbox, TF-IDF, CountVectorizer (CV), Word2Vec, FastText, VGG16 with XGB, stacked fusion models//hybrid stacked fusion model, PRS, AAO, KNN, decision tree, random forest, ANN, 3D-CNN model, Boruta FS algorithm, Gradient, Information Gain (IG), Multi-view Separable Pyramid Network (MiSePyNet), PyWinEA using Mono-objective and Multi-objective Genetic Algorithms (NSGAII), Elastic Net (EN), Gaussian Processes (GP), kNN, (LR), Linear Discriminant, Support Vector Machine, Voting classifiers, Multi-Classifier Network (MCN), Gradient Boosted Trees (GBTs), basic three-layer Neural Network architecture using the OASIS, Sparse K-means w/Resampling, a deep neural network architecture, Adaboost, graph convolutional and recurrent neural network (graph-CNN-RNN), Single hidden layer neural network, Single-layer bidirectional, LSTM, Three-layer CNN, Deep Belief Network (DBN), stacked auto-encoder (SAE), SVR, SVC, PLSR, Shallow Models, Feature Pyramid Network (FPN) and temporally structured SVM (TS-SVM).

Studies in our review suggested different algorithms for best accuracy to early detection of Alzheimer’s disease but Deep learning models especially CNNs, and SVM reported more effective than others. SVM and CNN each offer distinct advantages and limitations. SVM is advantageous due to its reliable classification accuracy and specificity, reaching about 93% accuracy and 87% sensitivity in some studies, making it efficient for handling smaller datasets with feature selection methods. However, it can struggle with high-dimensional data unless combined with dimensionality reduction techniques. In contrast, CNN models have shown high sensitivity and specificity in early detection of Alzheimer’s disease, with some studies reporting accuracies above 95%. Their advantages include high accuracy, automated feature extraction, and the non-invasive nature of using medical imaging data. However, these models require significant computational resources, large amounts of labeled data, and are often considered “black boxes” due to their lack of interpretability.

### Mild cognitive impairment (MCI)

ML algorithms used for MCI included 3D-CNN, support vector machines, Gaussian Naive Bayes(GNB), EMCI identification framework, LASSO logistic regression, Naïve Bayes, Decision Tree, RF, Gaussian, Polynomial-kernel Support Vector Machines, kNN, LR, Adaboost and TS-SVM model. Except for LASSO logistic regression, all showed remarkable performance in early detection of the disease and its development to AD and dementia.

For the early detection of MCI, 3D-CNNs have proven to be highly effective, with studies demonstrating over 95% accuracy, high sensitivity and specificity. The advantages of CNNs include their ability to automatically extract relevant features from complex datasets and their non-invasive application of medical imaging. However, they require substantial computational resources and large amounts of labeled data, and their decision-making processes are often not easily interpretable, rendering them “black boxes.” SVMs are another viable option, offering moderate sensitivity and good interpretability. However, they necessitate extensive parameter tuning and may overlook fine spatial features critical for accurate diagnosis. RF and Decision Trees provide high interpretability and effectively manage non-linear data, though they typically exhibit lower sensitivity than CNN models. Ensemble methods, such as combinations of LASSO logistic regression and Naïve Bayes, offer a balanced approach in terms of sensitivity and specificity. These methods can serve as cost-effective options for initial screenings, particularly when integrated with clinical biomarkers. Overall, the choice of model should consider the trade-offs between performance, interpretability, and resource requirements to optimize early detection of MCI.

Random forest, LR, support vector machine, LightGBM, kNN, Decision tree, Gaussian Naïve Bayes (gNB), Auto-sklearn, Gaussian Processes (GP) regression, Gaussian Process regression (GPR), CNN, Adaboost, NN and LDR algorithms were also successfully used for detection and progression of Huntington’s disease, Multiple Sclerosis, Amyotrophic lateral sclerosis (ALS), Corticobasal Syndrome (CS), Neurofibromatosis type 1, Amyloid and Delirium.

## Discussion

In this study, we investigated 108 studies evaluating patients with neurological diseases for early detection using ML algorithms. This study showcases specific and statistically significant findings to illustrate the progress in the area and the prospective influence of these advancements on the global management of neurocognitive and neurodegenerative illnesses.

AI technologies can retrieve data from medical texts and generate diagnostic and prediction models using this data. An extensive collection of electronic medical records amassed over a considerable period can serve as the fundamental data for this form of research (13). Traditional diagnostic methods for PD diagnosis may misdiagnose because they evaluate small movements that are hard to classify. Early non-motor symptoms of PD may be minor and caused by other illnesses. Thus, these symptoms are typically missed, making early PD diagnosis difficult. ML algorithms have been used to classify PD and healthy controls or patients with comparable clinical presentations to overcome these issues and improve PD diagnosis and evaluation. Multiple ML-based computer-aided diagnosis and detection (CADD) systems have shown promise in identifying PD patients from healthy controls (14). Using preclinical indicators of non-motor symptoms, including sleep Behavior Disorder (RBD) and olfactory loss, CSF measures, and dopaminergic imaging to classify early PD and healthy normal Prashanth et al. found SVM classification near-perfect (15). Balaji et al. proposed a multi-class learning technique that differs from earlier machine learning approaches, which often focus on binary classification to identify the existence of PD. In contrast, the proposed approach can not only classify but also quantify the stages of PD (16).

Individuals afflicted with MCI typically experience a deterioration in cognitive abilities, which significantly affects their general health. Importantly, failure to promptly identify this illness by medical professionals can readily progress into dementia. Using artificial intelligence, a dimensional assessment technique may seamlessly combine classical neuropsychological measurements and facilitate the diagnosis of AD (17). Similarly, Raees et al. present an initial automated deep learning system that utilizes a large MRI dataset of normal and 111 patients to predict AD. By evaluating the effectiveness of SVM and DNN models, they demonstrate that Deep learning has a significant level of accuracy, ranging from 80 to 90%, in predicting AD (18). Goenka and Tiwari introduced a three-class CNN that utilizes three computational approaches for neuroimaging to classify AD. Their suggested model was empirically validated, demonstrating classification accuracies of 97.48, 96.62, and 86.49% for big, medium, and small patches, respectively (19). Artificial neural networks revealed an intricate correlation between cognitive state and auditory function that cannot be easily anticipated only by considering the cognitive differences between individuals with and without AD (20).

Zhao et al. reported that Support Vector Machines (SVM), which integrate short-term clinical and brain MRI data, show potential in predicting the course of MS illness and identifying individuals who would benefit from more aggressive treatment approaches (21). Similarly, Law et al. found that the possibility of disability in MS was most accurately predicted using non-parametric machine learning techniques. It also can select those with the highest and lowest progression risk for inclusion in secondary progressive M.S. (22). Concordantly, Zhang et al. found that computational approaches (Lesion Segmentation Toolbox) provide more accurate conversion predictions from CIS to MS than human visual analysis (23). Goyal et al. utilized a machine learning technique to predict MS by analyzing serum cytokines. Their findings indicate that the RF model achieved an accuracy of 91%, suggesting its potential for predicting MS using serum cytokine levels. Moreover, the RF model demonstrated a 70% accuracy in classifying MS patients into remitting and non-remitting categories (24). These data were similar to other studies (25–27).

This comprehensive systematic study of 108 articles includes papers that illustrate significant patterns in using artificial intelligence for early detection of neurological illnesses. Therefore, the authors confine their focus to presenting factual information on utilizing AI techniques in various tasks without evaluating the quality of these investigations. Further studies are required to evaluate additional aspects, such as new neuroimaging measurements and blood and genetic biomarkers. The utilization of predictive algorithms, as detailed in this study, may enhance the development of collaborative visualization and decision-making tools for physicians and patients, as previously explored in another research. Future research could focus on decreasing the number of attributes without compromising accuracy. The proposed strategies can also be extended to tackle other chronic disorders. When creating AI models for medical issues, it is advisable to use simple computational methods with the available datasets to make it easier to implement the predictive tool in healthcare settings and solve economic issues.

## Data Availability

The original contributions presented in the study are included in the article/supplementary material, further inquiries can be directed to the corresponding author/s.
